# Longevity-Associated Core Gut Microbiota Mining and Effect of Mediated Probiotic Combinations on Aging Mice: Case Study of a Long-Lived Population in Guangxi, China

**DOI:** 10.3390/nu15071609

**Published:** 2023-03-26

**Authors:** Rui-Ding Li, Wen-Xuan Zheng, Qin-Ren Zhang, Yao Song, Yan-Ting Liao, Feng-Cui Shi, Xiao-Hui Wei, Fan Zhou, Xiao-Hua Zheng, Kai-Yan Tan, Quan-Yang Li

**Affiliations:** 1 College of Light Industry and Food Engineering, Guangxi University, Nanning 530004, China; 2 Guangxi Zhuang Autonomous Region Institute of Product Quality Inspection, Nanning 530200, China

**Keywords:** long-lived seniors, core gut microbiota, network analysis, antioxidant

## Abstract

With an ageing population, healthy longevity is becoming an important scientific concern. The longevity phenomenon is closely related to the intestinal microflora and is highly complicated; it is challenging to identify and define the core gut microbiota associated with longevity. Therefore, in this study, 16S rRNA sequencing data were obtained from a total of 135 faecal samples collected as part of the latest sampling and pre-collection initiative in the Guangxi longevity area, and weighted gene co-expression network analysis (WGCNA) was used to find a mediumpurple3 network module significantly associated with the Guangxi longevity phenomenon. Five core genera, namely, *Alistipes*, *Bacteroides*, *Blautia*, *Lachnospiraceae NK4A136 group*, and *Lactobacillus*, were identified via network analysis and random forest (RF) in this module. Two potential probiotic strains, *Lactobacillus fermentum* and *Bacteroides fragilis*, were further isolated and screened from the above five core genera, and then combined and used as an intervention in naturally ageing mice. The results show a change in the key longevity gut microbiota in mice toward a healthy longevity state after the intervention. In addition, the results show that the probiotic combination effectively ameliorated anxiety and necrosis of hippocampal neuronal cells in senescent mice, improving their antioxidant capacity and reducing their inflammation levels. In conclusion, this longer-term study provides a new approach to the search for longevity hub microbiota. These results may also provide an important theoretical reference for the healthification of the intestinal microflora in the general population, and even the remodelling of the structure of the longevity-state intestinal microflora.

## 1. Introduction

The intestinal microflora has a vital function in the growth and development of the human body. Microecological interactions between different microbiota and their hosts profoundly influence the orderly performance of many of the body’s vital functions [1]. As the body ages, these interactions continue to play an important regulatory role through inflammation [2] and the gut–brain axis [3], which are factors that must not be overlooked for a long and healthy life [4]. Previous studies have demonstrated that the constitution of the intestinal microflora of long-lived older adults (aged 90 years or over) differs from that of younger people [5,6]. It has also been found that the intestinal microflora of long-lived older adults is, in some sense, more effective in maintaining host health [7]. For this reason, the structural properties of the intestinal microflora of long-lived older adults are well worth studying. Some researchers have found that long-lived elderly people have a greater structural diversity of intestinal microflora than younger people [8]. Furthermore, the abundance structure of the intestinal microflora of long-lived older adults people undergoes age-related changes [9]. It is enriched with a more characteristic gut microbiota that benefits health such as *Akkermansia muciniphila*, *Bacteroides fragilis* and *Parabacteroides merdae* [10,11]. These studies have also attempted to identify longevity-associated traits in long-lived individuals using variations in the abundance of strains in the intestinal microflora. However, they have had to contend with geographical and ethnic differences, making it more difficult to find longevity-associated traits based on abundance alone. *Bifidobacterium* has been reported to be enriched in long-lived older people in Sardinia [10], while Emilia Romagna showed a decrease in the longevity group [12]. However, quantity is not everything, and the role of characteristic bacteria present at a lower abundance is not necessarily minor. For example, *Alistipes finegoldii* and *Alistipes putredinis* are not very abundant in human intestinal bacteria but are closely linked to human health conditions [13]. Under the consensus that the intestinal microflora is closely linked to human health, these particular issues present a challenge in respect of characterising the relationship between the longevity phenomenon and key gut microbiota. Previous studies, including by our team, have identified a reconfiguration of the intestinal microflora in long-lived older adults in Guangxi, China, and have sought characteristic microbiota in terms of abundance changes [14,15]. Researchers have also conducted analyses of the structural composition of the abundance of this intestinal microflora and their variations, but further exploration of them is still necessary given the complexity of interactions between intestinal microflora and hosts.

Several researchers have used WGCNA and RF to explore the interactions between intestinal microflora and the host phenotype to address the variability and complexity described above. From these studies, key phenotypically relevant gut microbiota have been identified. Some researchers have used WGCNA to identify *Ruminococcaceae UCG-002* as the core gene responsible for the development of food allergies in children [16]. Vernocchi et al. [17] used WGCNA to identify key gut microbes and associated metabolic markers for non-small cell lung cancer patients. Some researchers have used RF to differentiate the top 10 core gut microbes in children with tic disorders from healthy children [18], and Pan et al. [19] used RF to identify the top 11 core gut microbes that could be used to differentiate schizophrenia. It is clear from the above that WGCNA and RF are often used for mining and differentiating the core gut microbiota. However, using more than one method for analysis may be more susceptible to noisy samples. Some researchers have found that by combining RF with other algorithms, RF can use the mechanism of multiple tree voting to cancel out some noise [20]. However, studies have yet to report combining RF and WGCNA to better analyse and mine the intestinal microflora. The use of WGCNA in combination with RF has the advantage of both mining key gut bacteria associated with the host phenotype and effectively distinguishing core groups of these gut bacteria, and it is therefore promising.

In terms of studying the regulatory effects of potential intestinal probiotics, Ramasamy et al. [21] used a series of methods, such as linear discriminant analysis, to identify five key gut microbes that regulate vitamin D deficiency. In addition, Wang et al. [22] used correlation analysis of intestinal microflora with levels of inflammatory factors to find that *Bifidobacterium pseudocatenulatum* may be an especially beneficial bacterium for improving impaired neurological and immune function. Although these studies used data analysis methods to identify some of the potential regulatory effects of intestinal probiotics, no targeted isolations of the characteristic gut microbiota have been cultured, and their actual regulatory effects have not been tested. Therefore, if we can isolate potential key probiotic strains from long-lived elderly people under the guidance of the key gut microbiota information obtained through a series of data analyses, we can determine how the intestinal microflora interacts with human health and longevity. The anti-ageing effect of these strains can then be effectively verified by using them on experimental animals and testing them for relevant indicators; this has not yet been reported.

Based on the above, in this study, we analysed the association between intestinal microflora and longevity traits by combining WGCNA with RF and other methods to construct different scale-free co-expression network modules from the obtained gut microbiota dataset of the Guangxi longevity population. An attempt was made to identify the pivotal network microbiota and explore those potential core microorganisms (strains) that are relatively less abundant but can regulate the longevity health of the host. These target strains were then screened from the faeces of the long-lived older adults and then applied to test animals to validate the probiotic effect of the newly screened strains. This study is intended to provide a new theoretical reference and regulatory pathway for healthy gut microbiota in the general population.

## 2. Materials and Methods

### 2.1. Sources of Volunteers and Their Basic Information

A total of 62 people from the longevity area (LA) of Donglan County, Guangxi, were recruited for this study, including 27 people aged 90 years or older. A questionnaire was administered to volunteers before the sample information was collected, and a rigorous selection process was carried out with the following inclusion criteria: all subjects (i) were in good health with no apparent disease and a body mass index (BMI) of 16.24–25.38 kg/m^2^; (ii) lived in a rural area with no additional dietary intervention; (iii) had not received medical care or been treated with probiotics, prebiotics, or antibiotic-related medications within the six months prior to the study. The collection of stool samples from volunteers was standardised and samples were immediately stored in ice boxes as follows: stool was collected in sterile lyophilised tubes with ice boxes, then transferred to −20 °C vehicle-mounted refrigerators (Ice Tiger A30, Guangzhou, China). Samples were then transported to the laboratory within 12 h for storage at −80 °C. In addition, the analysis of this study was combined with data from previous studies by the team [14,15]. In order to exclude possible effects due to geographical bias, only data from longevity villages in Donglan County, Guangxi, or neighbouring villages were included in this study, and the above inclusion criteria were met. Ultimately, this study was based on raw 16S rRNA sequencing data from 135 healthy long-lived population stool samples. 16S rRNA data from a total of 23 healthy population stool samples from urban non-long-lived area (NLA) in Nanning, Guangxi, with a mean age of 62.86 ± 8.17 years, were selected as controls, as shown in Table 1. Information regarding the volunteers is shown in Supplementary Table S1.

### 2.2. Microbial DNA Extraction and Illumina Mise Sequencing

The total DNA of bacteria in feces was extracted using the MagPure Stool DNA KF kit B kit (Shenzhen Magen Technology Co., Ltd., Shenzhen, China) and quantified using a Qubit fluorometer corresponding to DNA. The quality of the extracted DNA was detected by 1% agarose gel electrophoresis. The PCR reaction conditions were 94 °C for 3 min (pre-denaturation), followed by 30 cycles at 94 °C for 30 s (denaturation), 56 °C for 45 s (annealing), 72 °C for 45 s (extension), and a final extension of 10 min at 72 °C. The PCR products were purified using AmpureXP magnetic beads. Libraries were quality-checked and sequenced on the IIIumina MiSeq platform (BGI, Shenzhen, China) according to IIIumina’s standard procedure to generate two × 300 bp double-end sequences. All raw sequencing data were processed using the microbiome module in the Biomedical Genomics Workbench, a histology data-processing software. The low-quality-expressed data were first filtered out, and the quality-controlled data were compared with the SilvaSSURef_123_NR database using Qiime software. Sequences were then aggregated into the corresponding operational taxonomic units (OTUs) at a 97% similarity level, and the OTUs’ richness profiles were generated by sequence number.

### 2.3. Bioinformatic Analysis

#### 2.3.1. Characterisation of the Intestinal Microflora

Alpha diversity indices, including the observed species index, Simpson, Chao1, and Shannon indices, were calculated for each sample using Qiime software. Venn diagrams were plotted using the “VennDiagram” package in R software (version 4.0.1) and pooled diagrams were plotted using the “UpsetR” package. In addition, PCA plots of the principal components of each data set were plotted using species richness tables and analyzed using PERMANOVA tests to calculate the significance of differences between the groups. Box plots were plotted using the R software package “Ggplot2”, along with the corresponding histograms, subjected to Wilcoxon rank-sum tests.

#### 2.3.2. Building a Network of Microecological Co-Expression

We used the “WGCNA” package in R software to remove outlier OTU gene sequences and to construct a microecological co-expression network. Specifically, the pickSoftThreshold function was used to calculate the weighting factor β. The soft threshold power was selected based on approximating a scale-free topology and ensuring low average connectivity [23]. The adjacency matrix was calculated based on the soft threshold to construct the topological overlap matrix (TOM). Mean chain hierarchical clustering was performed based on the dissimilarity measure of the TOM, setting the minimum size of the gene dendrogram (genome) to 25 and setting the sensitivity to 3. The modules were then divided using the dynamic shearing algorithm, and the correlation between the modules and the explored phenotypes and significance were calculated from the phenotypic data. In addition, modules with distances less than 0.25 were merged, and it should be noted that the gray module was considered a collection of genes that could not be assigned to any module.

#### 2.3.3. Selection and Visualisation of Core Gut Microbiota

After calculating the correlation coefficients between modules and phenotypes, we use module feature correlation analysis to select significantly important modules with *p*-values of <0.05. The taxa of significant modules were visualised using Cytoscape (version 3.8.2) [24]. In addition, we used the cytoHubba plugin in Cytoscape to obtain core OTUs in the intercrossing network and annotate them such as to determine core species associated with the traits studied.

#### 2.3.4. Functional Prediction Analysis

We use the “Tax4Fun” software package in the R software, select SILVA123 as the reference file, and map the annotated OTU list to the kyoto encyclopedia of genes and genomes (KEGG) path.

#### 2.3.5. Classification of Characteristic Intestinal Gut Microbiota

A random forest model containing 500 decision trees with a maximum depth of 7 was built using the random forest classifier function in the Python (version 3.0) environment. Using the RF model, it was possible to distinguish between the long-lived and non-long-lived groups of the highest taxa at the genus level.

### 2.4. Relative Expression Assay of Target Genera

The expression of target genera in faecal samples from LA was tested by qPCR using total bacteria in faecal samples as an internal reference gene and faecal samples from older people in NLA as controls, and we listed the primer sets in Supplementary Table S2. Each PCR mixture (20 μL) sample contained DNA (2 μL), SYBR Green qPCR Mix (10 μL), the corresponding forward and reverse primers (0.5 µL each), and RNase-Free ddH_2_O (7 μL). qPCR cycling conditions were pre-denaturation at 95 °C for 5 min, followed by 40 cycles of denaturation at 95 °C for 10 s; primer-specific annealing for 30 s (Supplementary Table S2), followed by 40 cycles of extension at 72 °C for 30 s; and melting dissociation curve analysis at the end of the PCR analysis. The relative expression of each group of strains was finally calculated by the method in the reference literature [25], all expression experiments were repeated, and significance was assessed by the 2^−ΔΔCT^ method.

### 2.5. Isolation and Basic Characterisation of the Strain

#### 2.5.1. Methods for the Isolation and Screening of Core Strains

Firstly, 1 g of the faecal sample (from a longevity population of Guangxi longevity area) was taken and graded using sterile phosphate buffer (PBS, pH = 7.0) to dilute the faecal sample. Samples at 10^−5^, 10^−6^, 10^−7^, and 10^−8^ dilutions were selected to be coated in sterilised modified de Man–Rogosa–Sharpe medium (MRS) solid medium (containing 0.8% CaCO_3_) and Reinforced clostridium medium (RCM) solid medium (supplemented with 5% pure sheep blood). The monocultures were then incubated at 37 °C for 36 h–56 h under anaerobic conditions. The monocultures with dissolved calcium and without hemolysis circles were isolated and purified by scribing for three consecutive generations. The purified colonies were further selected and inoculated in MRS and RCM liquid media for 16–18 h at 37 °C anaerobically for strain amplification. Finally, we performed 16S rDNA identification on the amplified bacterial fluid. The primers used for PCR were the universal primers for 16S rDNA, 27F for the upstream primer, and 1492R for the downstream primer (Supplementary Table S2). In addition, the reaction system and conditions used for PCR were shown in the Supplementary Tables S3 and S4, and the products were sent to Biotech Bioengineering (Shanghai) Co for identification. Based on the strain identification results, *Lactobacillus fermentum* and *Bacteroides fragilis* were selected for subsequent experiments.

#### 2.5.2. Simulated Gastrointestinal Transit Experiment

Slightly modified according to the method in the reference [26], the culture solution to be tested was centrifuged overnight (4000 r/min, 20 min) to collect the bacteria, washed twice with sterile normal saline, and suspended the bacterial weight in 10 mL of artificial gastric juice with a pH value of 2.5 (NaCl 0.002 g/mL, pepsin 0.0032 g/mL, adjust the pH value to 2.5 with 1 mol/L HCl, and filter for sterilisation). The bacteria were collected by centrifugation (4000 r/min, 20 min) and resuspended in 10 mL of artificial intestinal solution (KH_2_PO_4_ 0.0068 g/mL, Trypsin 0.01 g/mL, bile salts 0.003 g/mL, pH 8.0) at 37 °C, 200 r/min, and incubated for 120 min at 37 °C, 200 r/min. The number of viable bacteria in the samples before and after treatment was measured, and the corresponding survival rates were calculated (3 parallels).

#### 2.5.3. Determination of Self-Aggregation Capacity

Centrifuge the overnight culture solution of the bacteria to be tested (4000 r/min, 20 min), wash it with normal saline twice, and re-suspend the bacteria in PBS buffer solution so that the number of viable bacteria is 10^8^ CFU/mL. The bacterial suspension (5 mL) was subjected to vortex oscillation for 10 s and was kept at room temperature for 5 h. The absorbance values of the sample supernatant were measured at 600 nm initially (A_0_) and 5 h (A_5_). The following equation expresses the self-aggregation value:

self-aggregation value (%) = 1 − (A_5_/A_0_) × 100.

#### 2.5.4. Surface Hydrophobicity Testing

Centrifuge the culture solution to be tested overnight (4000 r/min, 20 min), wash it with normal saline twice, and resuspend it in 0.1 mol/L KNO_3_ (pH 6.2) so that its viable count concentration is 10^8^ CFU/mL and determine the absorbance A_0_ of the sample at 600 nm. Add 1 mL of xylene solvent to 3 mL of bacterial solution, leave at room temperature for 10 min, vortex shake the two-phase system for 2 min, leave at room temperature for 20 min, take the aqueous phase and measure the absorbance at 600 nm (A_1_). The formula expressed the percentage of bacterial adhesion to the solvent:

percentage adhesion (%) = 1 − (A_1_/A_0_) × 100.

#### 2.5.5. Comprehensive Quantitative Scoring of the Strains

The probiotic properties of the screened *Lactobacillus fermentum* and *Bacteroides fragilis* were ranked using principal component analysis concerning a comprehensive quantification method in the literature [27]. The three leading indicators chosen for this study were gastrointestinal tolerance, self-cohesion, and hydrophobicity. In addition, the data were standardised and dimensionless to eliminate the effect of differences in the size of the indicators. The overall quantitative score (*F*_sum_) was calculated using the following formula:

(1) $$F_{\mathrm{sum}}=\frac{v1}{M}\left(\sum_{k=1}^{2}a_{i}X_{i}\right)+\frac{v2}{M}\left(\sum_{k=1}^{2}b_{i}X_{i}\right)$$

where *vi* denotes the explained variance of each sample; *M* denotes the cumulative variance of the principal component analysis; *a_i_* and *b_i_* denote the first and second principal components, respectively; and *X_i_* denotes each sample’s standardised and dimensionless value.

### 2.6. Animal Experiments

#### 2.6.1. Experimental Strains

*Lactobacillus fermentum* LTP1332 and *Bacteroides fragilis* LTBF12 were isolated from the faeces of healthy centenarians in Guangxi longevity area and are stored in the School of Light Industry and Food Engineering, Guangxi University. Cells of LTP1332 and LTBF12 were then collected by centrifugation at 4000× *g* for 10 min at 4 °C and washed with phosphate buffered saline. After washing, LTP1332 and LTBF12 cells were resuspended in 0.9% saline to ensure a bacterial density of 1 × 10^9^ colony forming units (CFU)/mL for animal experiments.

#### 2.6.2. Experimental Animals

Twenty-month-old C57BL/6J naturally aged mice (SPF) were purchased from the Laboratory Animal Centre of Guangxi Medical University. All mice were individually housed in a 12:12 h light–dark cycle (temperature: 24 ± 2 °C, relative humidity: 55 ± 5%). Mice had free access to food and water. After one week of acclimatisation feeding, mice were randomly divided into three groups: control (C) mice were gavaged with sterile saline (same volume as used in the probiotic combination) daily; the low-dose (L) group was gavaged with 1 × 10^7^ CFU/mL *Lactobacillus fermentum* LTP1332 + 1 × 10^7^ CFU/mL *Bacteroides fragilis* LTBF12 daily; the high-dose (H) group was gavaged with 1 × 10^9^ CFU/mL *Lactobacillus fermentum* LTP1332 + 1 × 10^9^ CFU/mL *Bacteroides fragilis* LTBF12 daily. There were 12 males and females in each group, and treatment lasted for 6 weeks (Supplementary Figure S1).

#### 2.6.3. Open-Field Test Design for Mice

Each mouse was placed in a 100 × 100 × 30 cm^3^ open field chamber and explored for 5 min at liberty. A tracking camera was hung above the chamber to record the number of times the central area of the chamber was crossed, the total distance travelled, the number of times the mice stood upright on their hind limbs, and the number of times they groomed their hair. At the end of each mouse’s test, the mice were removed, the open field was cleaned of any faeces and urine left behind, and the field area was wiped with 75% alcohol.

#### 2.6.4. Brain Tissue Sectioning

After the behavioural experiment, all mice fasted for 12 h. After ether anaesthesia, brain tissue was rapidly removed by dissection on ice, rinsed in 0.9% physiological saline, blotted with residual water through filter paper, and fixed in 4% paraformaldehyde (Shanghai Li Rui Biological Technology Co., Ltd., Shanghai, China) for 48 h. The hippocampal region was observed under an inverted microscope (400×) after the samples were dehydrated, embedded in paraffin, sliced on a paraffin slicer, sectioned in a water bath, stained with HE, and sealed with neutral resin to reveal its morphological features.

#### 2.6.5. Quantification of Oxidation-Related Markers and Inflammatory Factors

Each brain tissue sample was homogenised in cold saline and centrifuged (1000× *g*, 4 °C, 20 min) to obtain a supernatant for further experiments. MDA, T-AOC, T-SOD, and GSH-Px oxidation markers in brain tissues were measured using the Mouse MDA Kit, T-AOC Kit, T-SOD Kit, and GSH-Px Kit (Nanjing Jiancheng Institute of Biological Engineering, Nanjing, Jiangsu, China). IL-6 and CRP were measured in hippocampal tissues using the Mouse IL-6 Kit and CRP Kit (Shanghai Jianglai Biotechnology Co., Ltd., Shanghai, China).

#### 2.6.6. qPCR Detection of Core Intestinal Microflora in the Mouse Intestine

Following the methods and primers mentioned in Section 2.4, the expression of the corresponding intestinal genera in the mouse gut before the intervention was used as the control; *Alistipes*, *Bacteroides*, *Blautia*, *Lachnospiraceae NK4A136 group*, and *Lactobacillus* were used to represent the relative expression of the target genera and were tested using qPCR.

## 3. Results

### 3.1. Characteristics of the Intestinal Microflora of the Long-Lived Population in Guangxi

**Figure 1:**
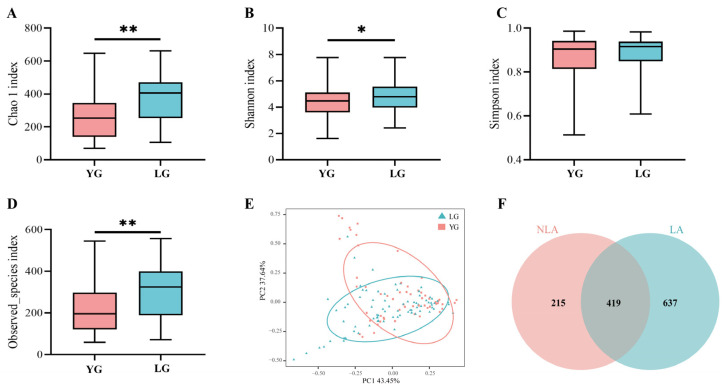
Characteristics of gut microbiota in Changshou District, Guangxi. (**A**–**D**) Comparison of α-diversity of intestinal microflora based on Chao1, Simpson, Shannon, and observed species index between LG and YG groups in Guangxi longevity area over 90 years of age. * indicates *p* < 0.05, and ** indicates *p* < 0.01 (Wilcoxon rank-sum test). (**E**) indicates principal component PCA analysis between LG longevity and YG groups. (**F**) OTU Venn diagram between longevity area (LA) and non-longevity area (NLA). (**F**) Ensemble plot showing the number of OTUs for each age group in the longevity area.

We divided the Guangxi-longevity-area-volunteer gut microbiota dataset into the LG longevity group, aged 90 years or older, and the YG young group, aged less than 90 years, and used Chao1, Simpson, Shannon, and the observed species index to assess alpha species diversity in the longevity area. Chao1 show that the LG longevity group contained more species in the sample compared to the YG group (*p* = 0.002) (Figure 1A) and that the LG group presented higher species richness (*p* = 0.006) (Figure 1D). The Shannon results indicated that the LG group had a higher abundance of gut microbial community composition than the YG group (*p* = 0.025) (Figure 1B). In addition, the Simpson results indicated that the LG group had a higher evenness of gut microbial community composition than the YG group, but the difference was not significant (Figure 1C). The results of the principal component analysis show that the gut microbiota structure of the LG longevity group is significantly different from that of the YG group (*p* = 0.003) (Figure 1E). The results from the non-intersection part of the Venn diagram show that the intestinal microbiota in the longevity area (LA) present greater specificity relative to the non-longevity area (NLA) (Figure 1F). In addition, the ratios of typical phylum in the centenarian (CE) to general elderly (GE) in the longevity area and the 70–86-year-old group in the non-longevity area (NLA) are shown in Supplementary Figure S2A,B. In particular, the ratio of *Firmicutes* to *Bacteroides* (F/B) was lowest in the CE group. The F/B values were significantly higher in the 70–86-year-old group in the non-longevity area (NLA) than in the GE group (*p* = 0.003) and the CE group (*p* < 0.001) (Supplementary Figure S2A). On the other hand, the ratio of *Firmicutes* to *Proteobacteria* (F/P) in the CE group was significantly lower than in the GE group (*p* = 0.002) and the 70–86-year-old group in the NLA (*p* = 0.002) (Supplementary Figure S2B).

### 3.2. Construction of Microecological Co-Expression Modules

**Figure 2:**
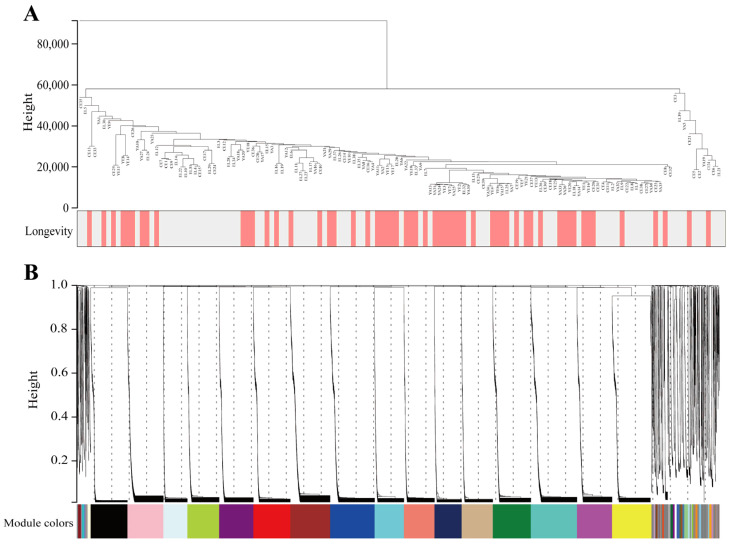
Construction of microecological co-expression modules. (**A**) Dendrograms and heatmaps of traits for 135 samples obtained from the longevity area. Colours indicate the proportion of traits, including longevity (age older 90 years) and sex. (**B**) Cluster dendrogram of OTUs. The branches above represent OTU clusters based on differences in topological overlap and the Colours of the assigned modules.

Co-expression networks were constructed using the WGCNA package in R software by separately calculating each gene sequence’s median absolute deviation (MAD), during which the top 50% of gene sequences with the smallest MAD were excluded. The microecological co-expression network was constructed using the above method by selecting 16,742 OTUs from sequences in 135 longevity-area population samples. The clustering analysis results of the samples are presented in Figure 2A. A soft threshold power was introduced in the network topology, reflecting the network’s scale independence and average connectivity. As can be observed in Supplementary Figure S3, when soft threshold *β* was chosen to be 14, the scale-free topology fit index *R*^2^ was close to 0.85, and the average connectivity tended to be close to 0. When *R*^2^ was close to 0.85, it indicated that the established network was closer to the scale-free network. When the average connectivity number gradually tended toward 0, it indicated that the average connectivity of the network was better [28]. In summary, it is suggested that the network built under this condition is closer to the scale-free network and that the network’s scale independence and average connectivity are good.

The filtered OTUs were hierarchically clustered using the topological overlap matrix to draw a clustering tree of similar overall distribution features that could be co-expressed, and then the generated clustering tree was cut using the dynamic shear tree algorithm. Following these operations, a total of 42 modules were identified, and OTUs with a high co-expression similarity were clustered into the same branch, with different branches of the cluster tree representing different modules, each of which was assigned a specific Colour (Figure 2B). The size of each module is presented in Supplementary Figure S4.

### 3.3. Identification of Core Network Modules and Visualisation Related to Longevity in Guangxi

**Figure 3:**
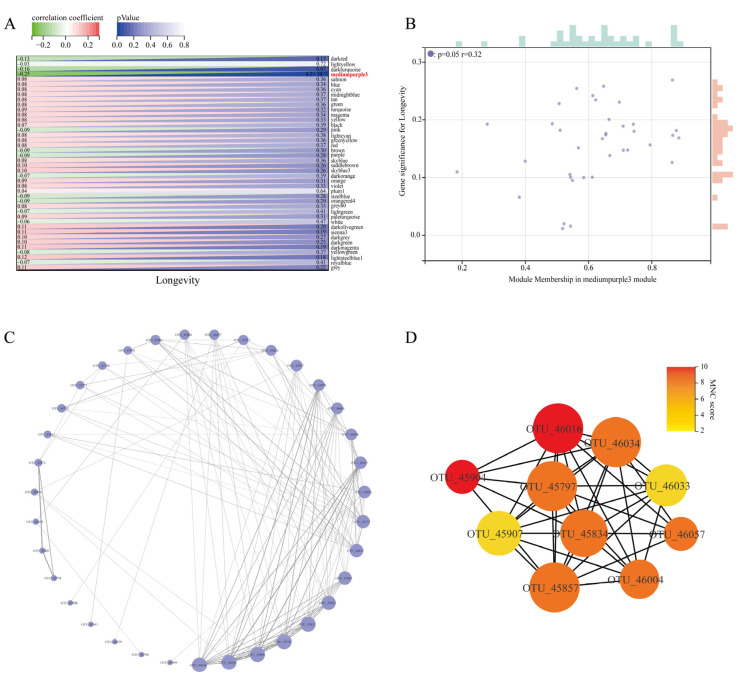
Identification of core network modules associated with longevity and visualisation. (**A**) Heatmap of trait correlations between modules. Each row corresponds to a trait module, and each column corresponds to a trait. The numbers on the left of the cells represent the correlation coefficient r-values, and the numbers on the right represent the *p*-values corresponding to each module. (**B**) Correlation between module membership (MM) and gene significance (GS) in the longevity key module is indicated. The GS scatter plot of longevity traits versus MM in the mediumpurple3 module is shown here. The size of the rectangle above and to the right of the image indicates the relative size of the number of genes at the same level. A larger rectangle indicates a larger relative size of the number of genes contained at that level. (**C**) Circular layout of the microecological co-expression network of the mediumpurple3 module. The size of the circles in the plot represents connectivity, and the darker colour of the connecting lines indicates higher weights. (**D**) Identification of core OTUs in the microecological co-expression network of the mediumpurple3 module using cytoHubba plugin. The size of the circles represents connectivity, and a darker node colour indicates a higher score for the maximum neighbour component (MNC), i.e., better centrality in the network module.

In order to find further intrinsic interactions between the co-expression modules, a correlation analysis of the trait data was conducted using the trait modules to determine the key modules associated with the longevity phenomenon, and the results are presented in Figure 3A. It was seen that the mediumpurple3 module co-abundance expression module was significantly correlated with the longevity trait (*r* = −0.25, *p* = 0.004). Further additional associations were identified between MM and GS for specific longevity traits, and it was found that there was a significant correlation between mediumpurple3 MM and GS for longevity traits (*r* = 0.32, *p* = 0.050) (Figure 3B). Based on these results, mediumpurple3 was identified as the key module for longevity traits in Guangxi for subsequent studies.

In order to identify further intrinsic interactions between the co-expression modules, a correlation analysis of the trait data was conducted using the trait modules to determine the key modules associated with the longevity phenomenon, and the results are presented in Figure 3A. It can be seen that the mediumpurple3 module co-abundance expression module was significantly correlated with the longevity trait (*r* = −0.25, *p* = 0.004). Further additional associations were identified between MM and GS for specific longevity traits, and it was found that there was a significant correlation between mediumpurple3 MM and GS for longevity traits (*r* = 0.32, *p* = 0.050) (Figure 3B). Based on these results, mediumpurple3 was identified as the key module for longevity traits in Guangxi for subsequent studies.

Functional enrichment analysis of this module revealed that all age groups were enriched in metabolism-related pathways, with the LG longevity group showing significantly higher lipid metabolism (*p* = 0.011) and metabolism of other amino acids (*p* < 0.001) than the YG group (Supplementary Figure S5a). The proportion of genes associated with genetic information processing increased gradually with age, peaking in the CE group. Overall, the LG longevity group had a lower proportion of genes in the human-disease-related pathway than the YG group, with significant differences in drug resistance: antimicrobial (*p* = 0.023); infectious disease: parasitic (*p* = 0.009); and infectious disease: viral (*p* = 0.042) (Supplementary Figure S5a). In addition, the association between significantly different second-tier metabolic pathways with relative abundance greater than 1% in the third-tier metabolic pathway and the genus was mined in the module (Supplementary Figure S5b). The genera found to be significantly associated with glycerophospholipid metabolism included *Alistipes* (*p* = 0.019), *Lachnospiraceae NK4A136 group* (*p* = 0.028), *Blautia* (*p* = 0.020), and *Turicibacter* (*p* = 0.031) (Supplementary Figure S5b).

In addition, R software was used to extract the edges and nodes in the mediumpurple3 network module. Combined with the connection weight and connectivity, the corresponding microbiological co-expression network (35 nodes, 122 edges) was constructed using Cytoscape (see Figure 3C for the results). We used the maximum neighbour component (MNC) in the cytoHubba plug-in to extract the top 10 core OTUs in the mediumpurple3 network and build the corresponding core OTU network (Figure 3D). The core OTUs in Figure 3D (Supplementary Table S5) include nine genera, namely, *Alistipes*, *Blautia*, *Bifidobacteriaceae*, *Bacteroides*, *Christensenellaceae R-7 group*, *[Eubacterium] coprostanoligenes group*, *Lachnospiraceae NK4A136 group*, *Lactobacillus*, *Ruminiclostridium 5*, and *Ruminococcaceae UCG-004*. The high centrality of these genera in the network also suggests that they can greatly influence the network structure of the microecological interactions in the mediumpurple3 module.

### 3.4. Identification of Core Genera in the Guangxi Longevity Core Module

**Figure 4:**
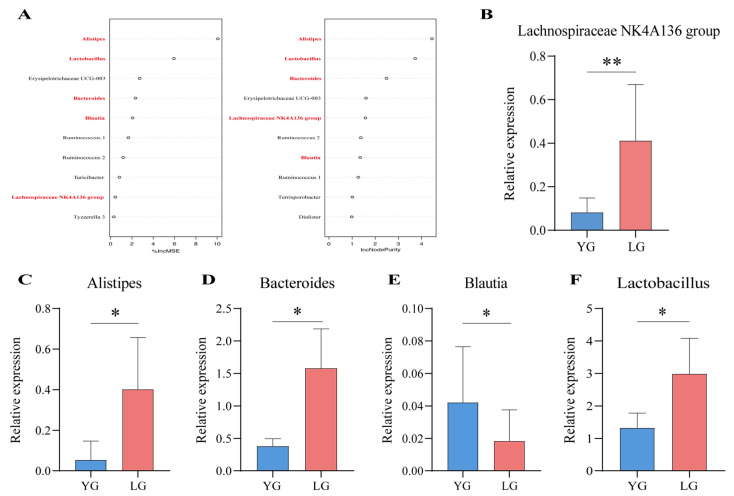
Random forests and differential gene expression identify key genera in the longevity core module. (**A**) Random forest prediction of iconic longevity taxa within the module and ranking of top 10 importance based on longevity phenomena, with taxa in bold indicating that they are also present in the top 10 results for network-centric colonies in the core module. On the left-hand side of the figure, the increase in mean-squared-error (IncMSE) ranking is indicated, and on the right-hand side the increase in node purity (IncNodePurity) is indicated, with higher values of %IncMSE as well as IncNodePurity indicating higher importance of the variable. (**B**–**F**) Results of qPCR relative expression tests for key gut microbiota, with both YG and LG groups using non-longevity area seniors as controls. (**B**) indicates *Lachnospiraceae NK4A136 group*, (**C**) indicates *Alistipes*, (**D**) indicates *Bacteroides*, (**E**) indicates *Blautia*, and (**F**) indicates *Lactobacillus*. * indicates *p* < 0.05 and ** indicates *p* < 0.01.

To further validate the core microbiota in the mediumpurple3 module, we distinguished the OTU-annotated enterobacterial genera in the module for the LG longevity group and the YG group using random forest modelling. The results show that *Alistipes*, *Lactobacillus*, *Erysipelotrichaceae UCG-003*, *Bacteroides*, *Blautia*, *Ruminococcus 1*, *Ruminococcus 2*, and *Lachnospiraceae NK4A136 group* appeared in the top 10 results of the two importance ranking of random forests (Figure 4A). In addition, *Alistipes*, *Bacteroides*, *Blautia*, *Lachnospiraceae NK4A136 group*, and *Lactobacillus* in the above results intersect with the top 10 of the network centre gut microbiota in the WGCNA results. Therefore, these five genera were selected as the key gut microbiota in respect of the longevity phenomenon in Guangxi and were further verified using qPCR. The results show that the relative expression of *Alistipes* (*p* = 0.048), *Bacteroides* (*p* = 0.011), *Lachnospiraceae NK4A136 group* (*p* = 0.004), and *Lactobacillus* (*p* = 0.043) was higher in the LG group than in the YG group (Figure 4B–D,F). In contrast, the relative expression of *Blautia* (*p* = 0.019) in the LG group was lower than that of the YG group (Figure 4E). We found the relative expression of the key microbiota to be significantly different between the LG and YG groups, which also validated the prediction above, thus pinpointing the five core genera of the gut microbiota of older adults living in Guangxi: *Alistipes*, *Bacteroides*, *Blautia*, *Lachnospiraceae NK4A136 group*, and *Lactobacillus*.

### 3.5. Results of the Combined Quantitative Scoring of the Probiotic Properties of the Strains

To further test the key role of core genera in influencing longevity and ageing, some strains were isolated from the faeces of centenarians (CE) and the general elderly (GE) in the Guangxi longevity area in this study. Wang et al. [22] found that *B. fragilis* was abundant in the intestines of centenarians and may influence healthy longevity through the inflammatory disease pathway. Meanwhile, Zhang et al. [29] found that *B. fragilis* could improve ageing-related atrial fibrillation in rats through the immune pathway. In addition, strains of *Bacteroides*, including *B. fragilis*, are considered the “next generation of probiotics” and could be used in dietary supplements as a potential strategy to promote human health [30]. On the other hand, Wu and colleagues [31] found that *Lactobacillus* in the intestine of centenarians helped build an antioxidant system, thus promoting a long and healthy life. Park et al. [32] found that among 18 lactic acid bacteria obtained from screening in Korean long-lived elderly, *Lactobacillus fermentum* showed better potential probiotic properties. In addition, Hor et al. [33] found that *Lactobacillus fermentum* could better lighten inflammation in ageing rats and has the potential for anti-ageing therapy. Therefore, we were guided by the key genera in this study and considered the above background. *Lactobacillus fermentum* and *Bacteroides fragilis* were deemed worthy of focused research exploration. To this end, the basic properties of the strains screened (simulated gastrointestinal tolerance, self-cohesion, and hydrophobicity) were first tested and analysed, with the results shown in Supplementary Table S6.

A Kaiser–Meyer–Olkin (KMO) test, based on the three probiotic indicators in Supplementary Table S6, yielded a KMO value of 0.639 > 0.6, indicating that a combined quantitative principal component score was possible [34]. Following a cumulative variance more significant than 90%, this study extracted two principal components, at which point the cumulative variance reached 93.137%. By bringing the standardised dimensionless data and the values of the loading matrix (Table 2) into Equation (1), the composite quantitative score for each strain can be calculated (Table 3). As can be seen from Table 3, *Lactobacillus fermentum* LTP1332 from centenarian faeces had a higher combined quantitative score than other *Lactobacillus fermentum* strains, and *Bacteroides fragilis* LTBF12 from centenarian faeces had a higher combined quantitative score than other *Bacteroides fragilis* strains. The above suggests that *Lactobacillus fermentum* LTP1332 and *Bacteroides fragilis* LTBF12 have good probiotic potential and could be made into a probiotic complex to intervene in naturally ageing mice.

### 3.6. Open-Field Experiments

**Figure 5:**
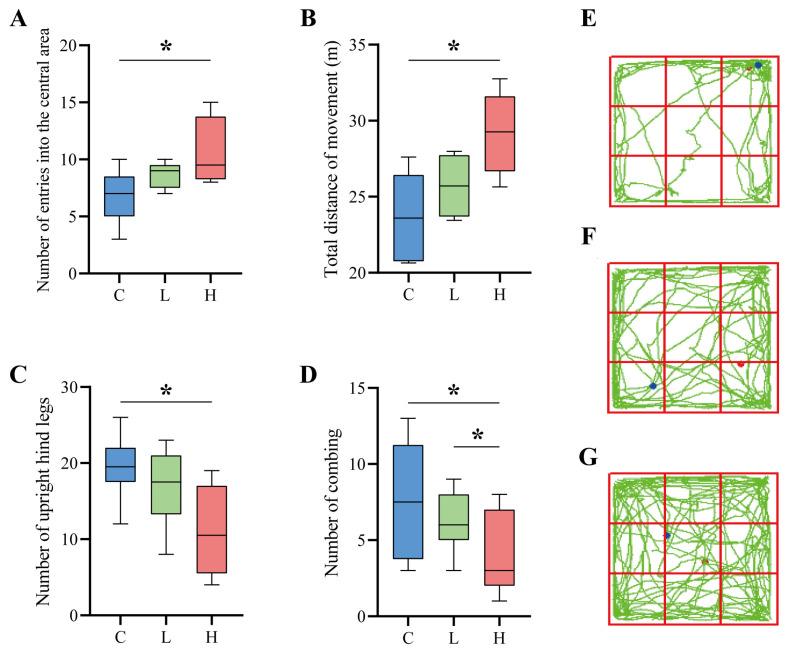
Results of the open-field experiment. (**A**–**D**) Parameters of the open field experiment. (**A**) indicates the number of times the mice entered the central area of the open field, (**B**) indicates the total distance the mice moved in the open field, (**C**) indicates the number of times the mice stood upright on their hind limbs during the open field experiment, (**D**) indicates the number of times the mice groomed during the open-field experiment; * indicates *p* < 0.05. (**E**–**G**) In the open field experiment, there are behavioural pathways of mice, with red dots in the diagram indicating the starting point and blue dots showing the endpoint. (**E**) denotes control group C, (**F**) denotes low-dose group L, and (**G**) denotes high-dose group H.

After six weeks of intervention, the general phenotype of the naturally aged mice showed that the mice with the probiotic combination had shinier hair and softer skin and were more mobile, active, and curious. In contrast, the control mice had sparse hair, dry skin, reduced physical activity, slow movement, narrow range of motion, and slow response. As shown in Figure 5A–D, the open-field experiment assessed the spontaneous exploration of new environments, locomotor ability, and anxiety resistance of naturally aged mice. Compared with the C group, the composite probiotic intervention group mice passed through the open-field experimental centre more times. Among them, the high-dose composite probiotic intervention group H showed a significant difference, and the number of passes increased by 52.42% (*p* = 0.036) compared with the control group C (Figure 5A), which indicates that the composite probiotic intervention can improve the spontaneous motor exploration ability of ageing mice. The total movement distance of mice in the open field experiment reflects the movement ability of mice. The total distance travelled by the mice in the open-field experiment reflects the locomotor ability of the mice. As shown in Figure 5B, the total distance travelled by the mice after the probiotic complex intervention increased, reflecting the improved ability of the mice after the intervention, with a significant increase of 22.97% in the total distance travelled by the high dose of the probiotic complex intervention group (*p* = 0.012). In addition, the number of times mice stood upright on their hind legs and the number of grooming sessions indicated the anxiety of the experimental animals, with a higher number of hind-limb standing incidents and grooming sessions indicating higher anxiety. As seen in Figure 5C, the number of hind-limb standing incidents decreased by 12.74% in the low-dose L group and by 43.95% in the H group (*p* = 0.015). In addition, the number of posterior grooming incidents was reduced by 17.58% in the L group and 49.09% in the H group (*p* = 0.014) (Figure 5D). Therefore, it is clear from the above that the probiotic complex intervention significantly improved the anxiolytic ability of the mice. As seen in Figure 5E, the control mice always moved along the perimeter of the open field with a single range of movement. Conversely, the mice with the probiotic complex intervention exhibited a broader range of movement in the field. In addition, they entered the centre of the open field several times (Figure 5F,G), indicating that the mice were in a relatively good emotional state, with a lower level of anxiety and a higher activity level compared to the control mice.

### 3.7. Probiotic Combinations Reduce Inflammation and Oxidative Stress in Naturally Ageing Mice

**Figure 6:**
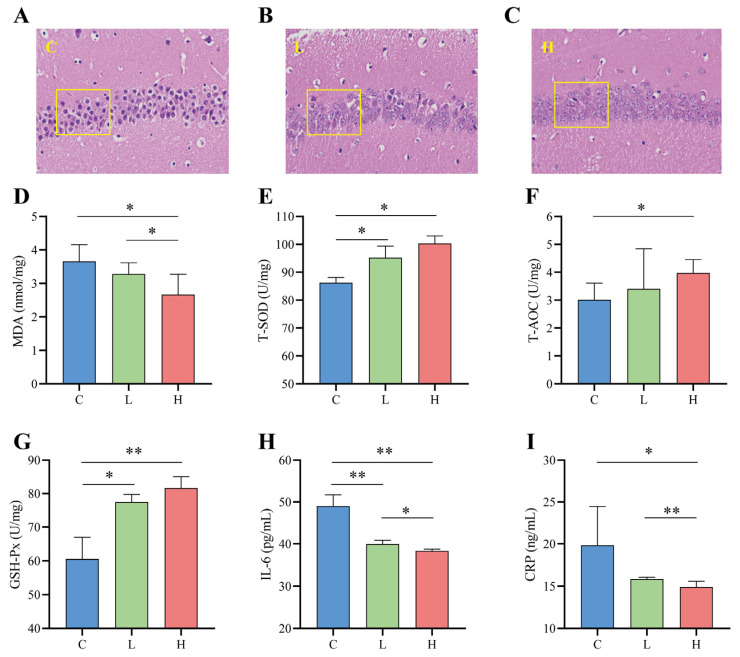
Effects of probiotic combinations on inflammation and oxidative stress in the brains of naturally aged mice. (**A**–**C**) Pathological sections (400×) of the CA1 region of the hippocampus of each group of mice, where (**A**) indicates control group C, (**B**) indicates low-dose intervention group L, and (**C**) indicates high-dose intervention group H. (**D**–**F**) Effect of the complex probiotics on antioxidant activity in the brains of naturally aged mice, showing the changes in MDA (**D**), T-SOD (**E**), T-AOC (**F**), and GSH-Px (**G**). (**H**,**I**) Changes in the effect of probiotic combinations on brain inflammation in naturally aged mice results show changes IL-6 (**H**) and CRP (**I**). * indicates *p* < 0.05; ** indicates *p* < 0.01.

The hippocampus is part of the limbic system of brain tissue and is directly involved in learning and memory processes. The CA1 region is closely related to the physiological functioning of the hippocampus. It is most sensitive to external pathological factors and is the site of the earliest pathological changes, so the hippocampal CA1 region is often used as a typical region for indicating brain injury [35]. Figure 6A–C show the microscopic structure of the hippocampal CA1 region of the mice in this test. As seen from the sections, the control L group had poorly differentiated pyramidal nerve cells in the hippocampal CA1 region, abnormal nerve cell morphology, smaller nuclei, and severe apoptosis (Figure 6A). The nuclei of the high-dose H group were more clearly visible, and the cell layers were more numerous and more closely ordered than those of the low-dose L group (Figure 6B,C).

Compared to the control C group, the probiotic combination intervention significantly enhanced the activity of total superoxide dismutase (T-SOD) (L and C, *p* = 0.027; H and C, *p* = 0.018), total antioxidant capacity (T-AOC) (H and C, *p* = 0.045), and glutathione peroxidase (GSH-Px) (L and C, *p* = 0.021; H and C, *p* < 0.001) in the brains of naturally senescent mice (Figure 6E–G), while decreasing the activity of malondialdehyde (MDA) (L and C, *p* = 0.045; H and C, *p* = 0.027) (Figure 6D). Furthermore, compared to the L group, the H group showed better antioxidant capacity T-SOD (10.46% increase in the L group and 16.36% increase in the H group), T-AOC (13.22% increase in the L group and 31.93% increase in the H group), and GSH-Px (25.45% increase in the L group and 39.45% increase in the H group) activities as well as lower MDA (L 10.43% lower and 27.22% lower in group H) activity. In addition to oxidative stress, the inflammatory cytokines in interleukin-6 (IL-6) and C-reactive protein (CRP) also play a crucial role in the ageing process [36]. As shown in Figure 6H,I, the probiotic combination significantly reduced the release of the inflammatory cytokines IL-6 (L and C, *p* < 0.001; H and C, *p* < 0.001) and CRP (H and C, *p* = 0.014) compared to the control C group, and the reduction in inflammatory cytokines was greater in the high-dose group than in the low-dose group (IL-6, *p* = 0.016; CRP, *p* = 0.008).

### 3.8. Effectiveness of Probiotic Combinations on Key Gut Microbiota for Longevity in Guangxi

**Figure 7:**
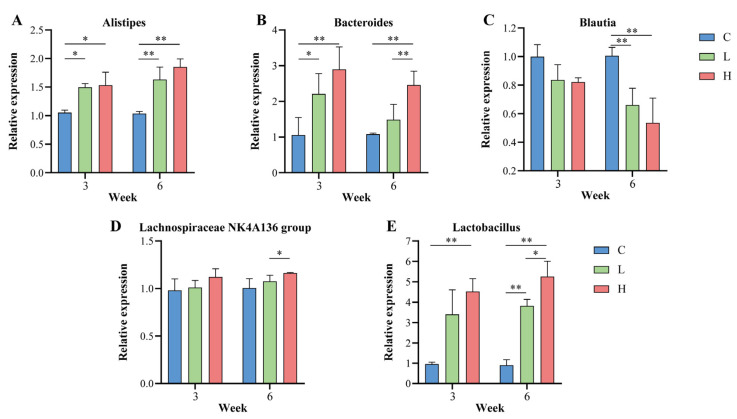
Effect of probiotic combinations on the core Guangxi longevity gut microbiota. (**A**–**E**) In this study, the intestinal genera of mice at the beginning of the intervention were used as controls to demonstrate the changes in the core longevity genera in the intestine of each group of mice at the middle (3 weeks) and end (6 weeks) of the probiotic combination intervention. (**A**) denotes *Alistipes*, (**B**) denotes *Bacteroides*, (**C**) denotes *Blautia*, (**D**) denotes *Lachnospiraceae NK4A136 group*, and (**E**) denotes *Lactobacillus*. * indicates *p* < 0.05; ** indicates *p* < 0.01.

To test the effect of probiotic combinations on the core genera of Guangxi longevity, in this study, qPCR relative expression was performed using the total gut microbiota as an internal reference gene; the corresponding intestinal genera in mice before the intervention as controls; and *Alistipes*, *Bacteroides*, *Blautia*, *Lachnospiraceae NK4A136 group*, and *Lactobacillus* as target genera. As shown in Figure 7A–E, the relative expression of *Alistipes*, *Bacteroides*, *Lachnospiraceae NK4A136 group*, and *Lactobacillus* increased gradually with increasing duration of the probiotic combination intervention. After six weeks of intervention, in the low-dose intervention group L, *Alistipes* significantly increased by 57.25% (*p* = 0.003), *Bacteroides* by 37.69% (*p* > 0.050), *Lachnospiraceae NK4A136 group* by 7.16% (*p* > 0.050), and *Lactobacillus* increased by 323.99% (*p* < 0.001). In the H group, *Alistipes* significantly increased by 78.63% (*p* < 0.001), *Bacteroides* significantly increased by 127.15% (*p* = 0.001), *Lachnospiraceae NK4A136 group* increased by 15.67% (*p* = 0.046), and *Lactobacillus* significantly increased by 482.42% (*p* < 0.001), with *Bacteroides* (*p* = 0.005), *Lachnospiraceae NK4A136 group* (*p* = 0.037), and *Lactobacillus* (*p* = 0.029) significantly higher in the H group than in the L group. However, *Blautia* gradually decreased as the duration of the probiotic combination intervention increased. After six weeks of intervention, *Blautia* was significantly reduced by 34.28% in the L group (*p* = 0.002), and *Blautia* was significantly reduced by 46.70% in the H group (*p* = 0.001).

## 4. Discussion

This study systematically analysed the characteristics of the intestinal microflora of 135 long-lived people in the Guangxi longevity area and built a technical, analytical framework with which to analyse the intestinal microbiota related to the longevity phenomenon by integrating WGCNA and RF methods. To address the challenges of the correlation between the intestinal microflora and longevity traits being influenced by multiple confounding factors [37,38] and key microbiota being difficult to define, we used this technique to discern five core genera associated with the longevity phenomenon in Guangxi and tested their practical effects.

Analysing the characteristics of the intestinal microflora of the long-lived older adults in Guangxi revealed that F/P showed a significant decline in the intestinal bacteria of the centenarians in Guangxi. In a similar study, Kim et al. found that F/P values also showed a decrease in long-lived elderly [9,39]. Furthermore, it has been shown that even though higher abundances of *Proteobacteria* can reflect instability in the gut microbial community, the results of this instability can also be present in non-disease traits (e.g., infant birth or post-operative recovery) [40]. Thus, decreasing F/P values may be a positive feature of a long and healthy life. However, we also found that older people living longer than 90 years in the longevity area of Guangxi, China, had higher gut microbiota diversity and richness. Gut microbiota diversity and abundance have been reported to be health markers in older adults [7]. Lower gut microbiota diversity may cause physical frailty and cognitive decline [41]. In line with the results of the present study, Ngangyola et al. [42] had also found higher intestinal microflora diversity and abundance in long-lived older adults relative to younger people. In this rich and diverse intestinal microecosystem, some core available microbiota [43] play an important role in equipping the long-lived elderly with the ability to cope with complex environments. If these genera can be highlighted from the complex microecosystem, this will provide great theoretical guidance and may have practical applications.

In order to explore the intestinal microflora closely related to the longevity phenomenon in the longevity population of Guangxi, China, we used WGCNA to construct a weighted microecological interactions network associated with longevity. In recent studies on the intestinal microflora of long-lived older adults people, some researchers have used unweighted networks for analysis. Biagi et al. [10] found by constructing unweighted networks that *Akkermansia*, *Bifidobacterium*, and *Christensenellaceae* were enriched in Italian long-lived older people. Ngangyola et al. [42] found, by constructing an unweighted network at the level of the intestinal microflora genus (relative abundance >0.5%), that *Alistipes*, *Bifidobacterium*, and *Eggerthella* co-occurred in the centenarian network in India and elsewhere. Conversely, the younger group did not show this feature. From the results of these studies, it can be seen that the construction of unweighted networks can be used to identify characteristic gut microbiota with structural differences in abundance between the longevity group and the younger group. However, the correlation between these intestinal microflora and longevity remains ambiguous. In addition, the relationship between two nodes in a non-weighted co-occurrence network is expressed by presence or absence, which may lead to information loss [44]. Compared with a non-weighted co-occurrence network, WGCNA retains the continuity of connectivity of network nodes. In this study, we identified a network module (mediumpurple3) significantly associated with longevity in Guangxi using WGCNA. We mined this module for ten network-centred gut microbiota, including *Alistipes*, *Bifidobacterium*, and *Christensenellaceae*, which were mentioned in the results of the two unweighted network studies above. However, this central gut microbiota also includes some genera that are relatively lower in abundance (less than 0.2%) and have not been reported in longevity-related studies, namely, the *Lachnospiraceae NK4A136 group*, *Ruminiclostridium_5*, and *Ruminococcaceae UCG-004*. Therefore, it is considered that using WGCNA can enable more comprehensive identification of the intestinal microflora associated with longevity in Guangxi.

To further reveal the core gut microbiota associated with the longevity phenomenon in Guangxi, we used the core gut microbiota of the network annotated by identification in the mediumpurple3 network module to form an intersection with the validation results of RF. Five core genera highly associated with longevity phenomena were identified in the Guangxi longevity area, namely, *Alistipes*, *Bacteroides*, *Blautia*, *Lachnospiraceae NK4A136 group*, and *Lactobacillus*. *Alistipes* have been reported to be a key trait for longevity in different populations [42,45]. *Bacteroides* and *Blautia* differ significantly in abundance between elderly Estonians and the general population [7]. *Lactobacillus* has also been reported to be enriched in healthy older people [46]. However, no reports have been found of these five genera being highly relevant core genera for the longevity phenomenon simultaneously. In addition, the qPCR results show significant differences in the performance of these five genera between the long-lived older adults and the young elderly in Guangxi, suggesting that *Alistipes*, *Bacteroides*, *Blautia*, *Lachnospiraceae NK4A136 group*, and *Lactobacillus* may play an important role in the microbial community of the long-lived older adults in Guangxi in influencing longevity. In this study, KEGG functional enrichment analysis in the module revealed that glycerophospholipid metabolism significantly correlated with the *Alistipes*, *Blautia*, and *Lachnospiraceae NK4A136 group* of the core genus. There is a strong association between altered metabolism and the ageing process [47], and glycerophospholipid metabolism is a significant marker of some inflammatory diseases [48,49]. Thus, the significant association of core genera with glycerophospholipid metabolism also suggests that they may be involved in health through pathways such as inflammatory diseases and thus influence human ageing longevity.

In order to investigate the actual anti-ageing effects and interactions of the core genera of Guangxi longevity, we used a probiotic combination guided by the core genera to intervene in naturally ageing mice. The results show that the probiotic combination improved the morphology of neuronal cells in the hippocampus and reduced the anxiety of the ageing mice. It was shown that the improved morphology and damage to neuronal cells in the hippocampus reflected improved cognitive and memory abilities, especially in the CA1 region [25] In addition, cognitive and memory loss due to ageing is usually accompanied by increased anxiety [50]. Therefore, it is clear from the above that the probiotic combination improved cognitive deficits and decreased anxiety caused by ageing in mice. In terms of the characterisation of antioxidant mechanisms, we found that the probiotic combination increased T-AOC, T-SOD, and GSH-Px oxidative markers and decreased MDA oxidative stress markers in mice [51]. In addition, T-SOD and GSH-Px are important antioxidant enzymes in the body, and higher T-SOD and GSH-Px levels can result in better scavenging of free radicals to protect cells [25]. The lower the MDA, the lower the damage to the body by free radicals [52]. From the above, the intervention of this probiotic combination can slow down the accumulation of free radicals due to ageing and thus improve ageing.

Inflammatory ageing is seen as a factor that determines the rate and longevity of the ageing process in individuals. Inflammatory factors commonly used clinically concerning older people’s health include IL-6, CRP [53], and tumour necrosis factor-*α* [54], with IL-6 and CRP being the most commonly used to assess inflammatory status markers. IL-6 is a cytokine produced by immune cells that promotes inflammatory responses, regulates immune and neuroendocrine functions, and is strongly associated with vascular diseases [55,56]. CRP is an acute phase response protein produced by the body following stressful stimuli. It is the most sensitive marker of the systemic inflammatory response [57]. In addition, elevated CRP is associated with an increased risk of morbidity and mortality in older patients [34]. Therefore, reduced IL-6 and CRP can be used as good indicators of individualised ageing health, and the intervention with this complex probiotic can effectively improve inflammatory ageing in naturally ageing mice. A previous study found that the combination of *Bifidobacterium longum* and *B. animalis*, isolated from the faeces of older people living in Guangxi, reduced D-gal-induced neuroinflammation and oxidative stress in senescent mice [58]. Consistent with the present study, the combination of *Lactobacillus fermentum* and *Bacteroides fragilis* directed by the core genus Guangxi longevity improved oxidative stress and inflammatory senescence in naturally senescent mice, and no previous study combining *Lactobacillus* and *Bacteroides* for senescence has been performed. Furthermore, our results show that the high dose of the combined probiotic group performed the best in terms of oxidative stress and inflammatory cytokines, demonstrating a dose-dependent effect that enhanced the body’s antioxidant capacity and improved inflammatory senescence.

To test the effect of probiotic combinations on the core genera of Guangxi longevity, we tracked changes in the relative expression of the core genera in natural mice. The results show that the expression levels of *Alistipes*, *Bacteroides*, *Lachnospiraceae NK4A136 group*, and *Lactobacillus* continued to increase after supplementation with the probiotic combinations, while in contrast, the expression levels of *Blautia* continued to decrease. Interestingly, we found that this change corresponded to a change in the abundance of gut microbiota in Guangxi long-lived elderly, i.e., *Alistipes*, *Bacteroides*, *Lachnospiraceae NK4A136 group*, and *Lactobacillus* were enriched in the intestines of Guangxi long-lived elderly. In contrast, the relative abundance of *Blautia* was reduced in the gut microbiota of the elderly in Guangxi, and this difference was also verified using qPCR. For the enrichment of *Alistipes*, *Bacteroides*, *Lachnospiraceae NK4A136 group*, and *Lactobacillus* in the longevity group found in this study, reduced abundance of *Alistipes* has been reported to be associated with some cardiovascular diseases as well as some inflammatory diseases [13]. *Bacteroides* has been reported to be associated with neurodevelopment, with higher levels of *Bacteroides* improving cognitive and language development [59]; the *Lachnospiraceae NK4A136 group*, a butyrate-producing bacterium, has been found to maintain the integrity of the intestinal barrier in mice and is negatively associated with intestinal permeability [60]. It is also believed that the enrichment of *Lactobacillus* in the gut helps to promote host metabolism and nutrient absorption [61]. Furthermore, for *Blautia* to be reduced in the longevity group, *Blautia* has been reported to have metabolic activity beneficial to host health. However, some of these strains produce secondary bile acids, such as deoxycholic acid, which have pro-carcinogenic effects [62]. From the above, it can be seen that the core genera changed in the gut of naturally aged mice after the intervention that favoured the longevity state, which also suggests that the key genera of Guangxi longevity mined through WGCNA combined with RF have a strong ability to interact with each other and play important roles that can influence healthy longevity.

Although this study has shed some light on the association between gut microecological networks and longevity in Guangxi, it may also have some limitations. For example, although we applied a novel network analysis system to explore key groups of bacteria associated with longevity in Guangxi, including those with low relative abundance but high association with longevity, a larger sample size and sequencing data from the macrogenome are necessary for testing. In addition, as this is a cross-sectional study, the results do not directly show a causal relationship between gut microbiota and longevity. The results would be more convincing if they were verified by the further screening of more strains of bacteria for the experiment, setting up an unfunctional strain as a negative control and by considering potential sex effects.

## 5. Conclusions

In this study, WGCNA analysis of the gene sequencing data from 135 faecal samples from the Guangxi longevity population identified a mediumpurple3 module significantly associated with the Guangxi longevity phenomenon. We constructed a novel analytical framework, combining the microecological interaction network of the mediumpurple3 module and RF to identify and target five core genera associated with longevity in Guangxi, namely, *Alistipes*, *Bacteroides*, *Blautia*, *Lachnospiraceae NK4A136 group*, and *Lactobacillus*. Using qPCR, we found their relative expression to be significantly different between the long-lived older adults and young group. Further, by screening and intervening with pure cultures of naturally ageing mice, we isolated two strains of the core genus, i.e., *Lactobacillus fermentum* and *Bacteroides fragilis*. We found that this combination improved the morphology of hippocampal neurons and reduced anxiety in ageing mice. The combination also increased T-AOC, T-SOD, and GSH-Px oxidative markers and decreased MDA oxidative stress markers and IL-6 and CRP inflammatory markers. However, the expression of the core Guangxi longevity genera in the gut of ageing mice changed towards longevity after the intervention. In conclusion, this study provides a more comprehensive elucidation of longevity-related microbial interactions. It may also offer new ideas for exploring longevity-related pivotal gut microbiota.

## Figures and Tables

**Table 1:** Basic information and grouping of subjects.

	Number of Subjects	Age	Age Mean ± SD Range	Groups	Sample Source
Younger seniors (YS)	36	55–69	58.97 ± 3.69	Young group (YG)	2 samples from Wang et al. [14], 20 samples from Ren et al. [15], and 35 samples from this study
General elderly (GE)	21	71–86	77.48 ± 5.63		
Older seniors (OS)	39	90–100	94.10 ± 3.12	Longevity group (LG)	13 samples from Wang et al. [14], 38 samples from Ren et al. [15], and 27 samples from this study
Centenarians (CE)	39	101–118	104.71 ± 3.93		
Non-longevity area in guangxi (NLA)	27	53–82	62.86 ± 8.17	NLA	Ren et al. [15]

**Table 2:** Load factor table.

Name	Principal Component 1	Principal Component 2
Characteristic roots	2.270	0.524
Explanation of variance	75.66%	17.48%
Gastrointestinal tolerance	0.811	0.567
Self-cohesive capacity	0.862	−0.442
Hydrophobic rate	0.932	−0.085

**Table 3:** Combined score results.

ID	Sources of Strain Isolation	Composite Score	Rank
LTP1332	CE	1.7769	1
LTP1334	CE	1.6918	2
LTP1333	CE	1.2906	3
LTP1806	GE	1.1452	4
LTBF12	CE	0.5311	5
LTP1832	CE	0.3808	6
LTBF13	CE	0.1980	7
LTBF11	CE	−0.0547	8
LTP1848	CE	−0.4013	9
LTBF31	GE	−0.5541	10
LTP1805	GE	−0.7543	11
LTBF32	GE	−1.6483	12
LTBF34	GE	−1.7454	13
LTP1804	GE	−1.8564	14

## Data Availability

The corresponding author can provide the data analyzed in the present study upon request that is deemed reasonable.

## Supplementary Materials

The following supporting information can be downloaded at: https://www.mdpi.com/article/10.3390/nu15071609/s1, Figure S1: Diagram showing experimental design and animal groupings for mice; Figure S2: Structural characteristics of the microbiota; Figure S3: The selection process of a soft threshold; Figure S4: Size of each module; Figure S5: Functional prediction results. Table S1: Details of subjects in each of the categorical groups analyzed in this study; Table S2: Primers and conditions; Table S3: PCR reaction system for strain identification; Table S4: PCR reaction conditions for strain identification. Table S5: Top 10 in the mediumpurple3 network ranked by MNC method. Table S6: Basic characteristics of the strains.

## References

[1] Valdes A.M., Walter J., Segal E., Spector T.D. (2018). Role of the gut microbiota in nutrition and health. BMJ—Br. Med. J..

[2] Ghosh T.S., Shanahan F., O Toole P.W. (2022). Toward an improved definition of a healthy microbiome for healthy aging. Nature Aging.

[3] Cryan J.F., Dinan T.G. (2012). Mind-altering microorganisms: The impact of the gut microbiota on brain and behaviour. Nat. Rev. Neurosci..

[4] Tavella T., Turroni S., Brigidi P., Candela M., Rampelli S. (2021). The Human Gut Resistome up to Extreme Longevity. Msphere.

[5] Rampelli S., Soverini M., D’Amico F., Barone M., Tavella T., Monti D., Capri M., Astolfi A., Brigidi P., Biagi E. (2020). Shotgun Metagenomics of Gut Microbiota in Humans with up to Extreme Longevity and the Increasing Role of Xenobiotic Degradation. Msystems.

[6] Wu L., Zeng T., Zinellu A., Rubino S., Kelvin D.J., Carru C. (2019). A Cross-Sectional Study of Compositional and Functional Profiles of Gut Microbiota in Sardinian Centenarians. Msystems.

[7] Sepp E., Smidt I., Rööp T., Štšepetova J., Kõljalg S., Mikelsaar M., Soidla I., Ainsaar M., Kolk H., Vallas M. (2022). Comparative Analysis of Gut Microbiota in Centenarians and Young People: Impact of Eating Habits and Childhood Living Environment. Front. Cell. Infect. Microbiol..

[8] Kong F., Deng F., Li Y., Zhao J. (2019). Identification of gut microbiome signatures associated with longevity provides a promising modulation target for healthy aging. Gut Microbes.

[9] Kim B.S., Choi C.W., Shin H., Jin S.P., Bae J.S., Han M., Seo E.Y., Chun J., Chung J.H. (2019). Comparison of the Gut Microbiota of Centenarians in Longevity Villages of South Korea with Those of Other Age Groups. J. Microbiol. Biotechnol..

[10] Biagi E., Franceschi C., Rampelli S., Severgnini M., Ostan R., Turroni S., Consolandi C., Quercia S., Scurti M., Monti D. (2016). Gut Microbiota and Extreme Longevity. Curr. Biol..

[11] Wang N., Li R., Lin H., Fu C., Wang X., Zhang Y., Su M., Huang P., Qian J., Jiang F. (2019). Enriched taxa were found among the gut microbiota of centenarians in East China. PLoS ONE.

[12] Rampelli S., Candela M., Turroni S., Biagi E., Collino S., Franceschi C., O’Toole P.W., Brigidi P. (2013). Functional metagenomic profiling of intestinal microbiome in extreme ageing. Aging.

[13] Parker B.J., Wearsch P.A., Veloo A., Rodriguez-Palacios A. (2020). The Genus Alistipes: Gut Bacteria With Emerging Implications to Inflammation, Cancer, and Mental Health. Front. Immunol..

[14] Wang F., Yu T., Huang G., Cai D., Liang X., Su H., Zhu Z., Li D., Yang Y., Shen P. (2015). Gut Microbiota Community and Its Assembly Associated with Age and Diet in Chinese Centenarians. J. Microbiol. Biotechnol..

[15] Ren M., Li H., Fu Z., Li Q. (2021). Succession Analysis of Gut Microbiota Structure of Participants from Long-Lived Families in Hechi, Guangxi, China. Microorganisms.

[16] Lee K.H., Guo J., Song Y., Ariff A., O’Sullivan M., Hales B., Mullins B.J., Zhang G. (2021). Dysfunctional Gut Microbiome Networks in Childhood IgE-Mediated Food Allergy. Int. J. Mol. Sci..

[17] Vernocchi P., Gili T., Conte F., Del C.F., Conta G., Miccheli A., Botticelli A., Paci P., Caldarelli G., Nuti M. (2020). Network Analysis of Gut Microbiome and Metabolome to Discover Microbiota-Linked Biomarkers in Patients Affected by Non-Small Cell Lung Cancer. Int. J. Mol. Sci..

[18] Xi W., Gao X., Zhao H., Luo X., Li J., Tan X., Wang L., Zhao J.B., Wang J., Yang G. (2021). Depicting the composition of gut microbiota in children with tic disorders: An exploratory study. J. Child Psychol. Psychiatry.

[19] Pan R., Zhang X., Gao J., Yi W., Wei Q., Su H. (2020). Analysis of the diversity of intestinal microbiome and its potential value as a biomarker in patients with schizophrenia: A cohort study. Psychiatry Res..

[20] Guo S., Zhang H., Chu Y., Jiang Q., Ma Y. (2022). A neural network-based framework to understand the type 2 diabetes-related alteration of the human gut microbiome. iMeta.

[21] Ramasamy B., Magne F., Tripathy S.K., Venugopal G., Mukherjee D., Balamurugan R. (2021). Association of Gut Microbiome and Vitamin D Deficiency in Knee Osteoarthritis Patients: A Pilot Study. Nutrients.

[22] Wang J., Qie J., Zhu D., Zhang X., Zhang Q., Xu Y., Wang Y., Mi K., Pei Y., Liu Y. (2022). The landscape in the gut microbiome of long-lived families reveals new insights on longevity and aging—Relevant neural and immune function. Gut Microbes.

[23] Jiang F., Gao H., Qin W., Song P., Wang H., Zhang J., Liu D., Wang D., Zhang T. (2021). Marked Seasonal Variation in Structure and Function of Gut Microbiota in Forest and Alpine Musk Deer. Front. Microbiol..

[24] Li M., Li D., Tang Y., Wu F., Wang J. (2017). CytoCluster: A Cytoscape Plugin for Cluster Analysis and Visualization of Biological Networks. Int. J. Mol. Sci..

[25] Yu X., Liang X., Han K., Shi F., Meng N., Li Q. (2022). Anti-Aging Effect of Dietary Fiber Compound Mediated by Guangxi Longevity Dietary Pattern on Natural Aging Mice. Nutrients.

[26] Fernandez M.F., Boris S., Barbes C. (2003). Probiotic properties of human lactobacilli strains to be used in the gastrointestinal tract. J. Appl. Microbiol..

[27] Zheng W., Li R., Zhou Y., Shi F., Song Y., Liao Y., Zhou F., Zheng X., Lv J., Li Q. (2022). Effect of dietary protein content shift on aging in elderly rats by comprehensive quantitative score and metabolomics analysis. Front. Nutr..

[28] Zhang B., Horvath S. (2005). A General Framework for Weighted Gene Co-Expression Network Analysis. Stat. Appl. Genet. Mol. Biol..

[29] Zhang Y., Sun D., Zhao X., Luo Y., Yu H., Zhou Y., Gao Y., Han X., Duan Y., Fang N. (2022). Bacteroides fragilis prevents aging-related atrial fibrillation in rats via regulatory T cells-mediated regulation of inflammation. Pharmacol. Res..

[30] O’Toole P.W., Marchesi J.R., Hill C. (2017). Next-generation probiotics: The spectrum from probiotics to live biotherapeutics. Nat. Microbiol..

[31] Wu L., Xie X., Li Y., Liang T., Zhong H., Yang L., Xi Y., Zhang J., Ding Y., Wu Q. (2022). Gut microbiota as an antioxidant system in centenarians associated with high antioxidant activities of gut-resident Lactobacillus. NPJ Biofilms Microbomes.

[32] Park J.S., Shin E., Hong H., Shin H.J., Lee Y. (2015). Characterization of Lactobacillus fermentum PL9988 Isolated from Healthy Elderly Korean in a Longevity Village. J. Microbiol. Biotechnol..

[33] Hor Y.Y., Lew L.C., Jaafar M.H., Lau A.S., Ong J.S., Kato T., Nakanishi Y., Azzam G., Azlan A., Ohno H. (2019). Lactobacillus sp. improved microbiota and metabolite profiles of aging rats. Pharmacol. Res..

[34] Bernard D., Gosselin K., Monte D., Vercamer C., Bouali F., Pourtier A., Vandenbunder B., Abbadie C. (2004). Involvement of Rel/Nuclear Factor-κB Transcription Factors in Keratinocyte Senescence. Cancer Res..

[35] Lopez-Gonzalez I., Tebe C.C., Ferrer I. (2017). Regional Gene Expression of Inflammation and Oxidative Stress Responses Does Not Predict Neurodegeneration in Aging. J. Neuropathol. Exp. Neurol..

[36] Sayed N., Huang Y., Nguyen K., Krejciova-Rajaniemi Z., Grawe A.P., Gao T., Tibshirani R., Hastie T., Alpert A., Cui L. (2021). An inflammatory aging clock (iAge) based on deep learning tracks multimorbidity, immunosenescence, frailty and cardiovascular aging. Nat. Aging.

[37] Zhong X., Powell C., Phillips C.M., Millar S.R., Carson B.P., Dowd K.P., Perry I.J., Kearney P.M., Harrington J.M., O’Toole P.W. (2021). The Influence of Different Physical Activity Behaviours on the Gut Microbiota of Older Irish Adults. J. Nutr. Health Aging.

[38] Reimer R.A. (2018). Establishing the role of diet in the microbiota–disease axis. Nat. Rev. Gastroenterol. Hepatol..

[39] Park S.H., Kim K.A., Ahn Y.T., Jeong J.J., Huh C.S., Kim D.H. (2015). Comparative analysis of gut microbiota in elderly people of urbanized towns and longevity villages. BMC Microbiol..

[40] Shin N.R., Whon T.W., Bae J.W. (2015). Proteobacteria: Microbial signature of dysbiosis in gut microbiota. Trends Biotechnol..

[41] Verdi S., Jackson M.A., Beaumont M., Bowyer R., Bell J.T., Spector T.D., Steves C.J. (2018). An Investigation Into Physical Frailty as a Link Between the Gut Microbiome and Cognitive Health. Front. Aging Neurosci..

[42] Tuikhar N., Keisam S., Labala R.K., Imrat, Ramakrishnan P. (2019). Comparative analysis of the gut microbiota in centenarians and young adults shows a common signature across genotypically non-related populations. Mech. Ageing Dev..

[43] La-Ongkham O., Nakphaichit M., Nakayama J., Keawsompong S., Nitisinprasert S. (2020). Age-related changes in the gut microbiota and the core gut microbiome of healthy Thai humans. 3 Biotech.

[44] Horvath S. (2011). Weighted Network Analysis.

[45] Sato Y., Atarashi K., Plichta D.R., Arai Y., Sasajima S., Kearney S.M., Suda W., Takeshita K., Sasaki T., Okamoto S. (2021). Novel bile acid biosynthetic pathways are enriched in the microbiome of centenarians. Nature.

[46] Yu X., Wu X., Qiu L., Wang D., Gan M., Chen X., Wei H., Xu F. (2015). Analysis of the intestinal microbial community structure of healthy and long-living elderly residents in Gaotian Village of Liuyang City. Appl. Microbiol. Biotechnol..

[47] Lee S.H., Park S., Kim H., Jung B.H. (2014). Metabolomic approaches to the normal aging process. Metabolomics.

[48] Su J., Li S., Chen J., Jian C., Hu J., Du H., Hai H., Wu J., Zeng F., Zhu J. (2022). Glycerophospholipid metabolism is involved in rheumatoid arthritis pathogenesis by regulating the IL-6/JAK signaling pathway. Biochem. Biophys. Res. Commun..

[49] Carrard J., Gallart-Ayala H., Infanger D., Teav T., Wagner J., Knaier R., Colledge F., Streese L., Konigstein K., Hinrichs T. (2021). Metabolic View on Human Healthspan: A Lipidome-Wide Association Study. Metabolites.

[50] Bunce D., Batterham P.J., Mackinnon A.J., Christensen H. (2012). Depression, anxiety and cognition in community-dwelling adults aged 70 years and over. J. Psychiatr. Res..

[51] Mulabagal V., Lang G.A., Dewitt D.L., Dalavoy S.S., Nair M.G. (2009). Anthocyanin Content, Lipid Peroxidation and Cyclooxygenase Enzyme Inhibitory Activities of Sweet and Sour Cherries. J. Agric. Food Chem..

[52] Liang L.L., Cai S.Y., Gao M., Chu X.M., Sun K.L. (2019). Purification of antioxidant peptides of Moringa oleifera seeds and their protective effects on H2O2 oxidative damaged Chang liver cells. J. Funct. Food..

[53] Puzianowska-Kuznicka M., Owczarz M., Wieczorowska-Tobis K., Nadrowski P., Chudek J., Slusarczyk P., Skalska A., Jonas M., Franek E., Mossakowska M. (2016). Interleukin-6 and C-reactive protein, successful aging, and mortality: The PolSenior study. Immun. Ageing.

[54] De Martinis M., Franceschi C., Monti D., Ginaldi L. (2005). Inflamm-ageing and lifelong antigenic load as major determinants of ageing rate and longevity. FEBS Lett..

[55] Jenny N.S., French B., Arnold A.M., Strotmeyer E.S., Newman A.B. (2012). Long-term Assessment of Inflammation and Healthy Aging in Late Life: The Cardiovascular Health Study All Stars. J. Gerontol..

[56] Mooijaart S.P., Sattar N., Trompet S., Lucke J., Stott D.J., Ford I., Jukema J.W., Westendorp R.G., de Craen A.J. (2013). Circulating interleukin-6 concentration and cognitive decline in old age: The PROSPER study. J. Intern. Med..

[57] Zhang H., Yang F., Guo Y., Wang L., Fang F., Wu H., Nie S., Wang Y., Fung M.L., Huang Y. (2018). The contribution of chronic intermittent hypoxia to OSAHS: From the perspective of serum extracellular microvesicle proteins. Metab.-Clin. Exp..

[58] Xia C., Cao X., Cui L., Liu H., Wang S., Chen T. (2020). Anti-aging effect of the combination of Bifidobacterium longum and B. animalis in a d-galactose-treated mice. J. Funct. Food..

[59] Tamana S.K., Tun H.M., Konya T., Chari R.S., Field C.J., Guttman D.S., Becker A.B., Moraes T.J., Turvey S.E., Subbarao P. (2021). Bacteroides-dominant gut microbiome of late infancy is associated with enhanced neurodevelopment. Gut Microbes.

[60] Ma L., Ni Y., Wang Z., Tu W., Ni L., Zhuge F., Zheng A., Hu L., Zhao Y., Zheng L. (2020). Spermidine improves gut barrier integrity and gut microbiota function in diet-induced obese mice. Gut Microbes.

[61] Turroni F., Ventura M., Buttó L.F., Duranti S., Toole P.W.O., Motherway M.O.C., van Sinderen D. (2014). Molecular dialogue between the human gut microbiota and the host: A Lactobacillus and Bifidobacterium perspective. Cell. Mol. Life Sci..

[62] Liu X., Mao B., Gu J., Wu J., Cui S., Wang G., Zhao J., Zhang H., Chen W. (2021). Blautia-a new functional genus with potential probiotic properties. Gut Microbes.

