# Supplementary material for: Longevity-Associated Core Gut Microbiota Mining and Effect of Mediated Probiotic Combinations on Aging Mice: Case Study of a Long-Lived Population in Guangxi, China
Source: Nutrients. 2023 Mar 26;15(7):1609. doi: 10.3390/nu15071609 (PMC10097023; doi:10.3390/nu15071609)
Supplement: Supplementary file 1 [file nutrients-15-01609-s001.zip › nutrients-2298524-supplementary.pdf]

# Online Supplement for: Longevity-Associated Core Gut Microbiota Mining and Effect of Mediated Probiotic Combinations on Aging Mice: Case Study of a Long-Lived Population in Guangxi, China

Rui-Ding Li, Wen-Xuan Zheng, Qin-Ren Zhang, Yao Song, Yan-Ting Liao, Feng-Cui Shi, Xiao-Hui Wei, Fan Zhou, Xiao-Hua Zheng, Kai-Yan Tan and Quan-Yang Li

**Supplement Contents:**

**Table S1.** Details of subjects in each of the categorical groups analyzed in this study.

**Table S2.** Primers and conditions.

**Table S3.** PCR reaction system for strain identification.

**Table S4.** PCR reaction conditions for strain identification.

**Table S5.** Top 10 in the mediumpurple3 network ranked by MNC method.

**Table S6.** Basic characteristics of the strains.

**Figure S1.** Diagram showing experimental design and animal groupings for mice.

**Figure S2.** Structural characteristics of the microbiota.

**Figure S3.** The selection process of a soft threshold.

**Figure S4.** Size of each module.

**Figure S5.** Functional prediction results.

## Tables

**Table S1.** Details of subjects in each of the categorical groups analyzed in this study.

| Sample ID | Essential information | | | | Personal habits | | | | | | Geographical position | | |
|---|---|---|---|---|---|---|---|---|---|---|---|---|---|
| | Group | Age | Gender | BMI (kg/m²) | Probiotic treatment (within the first 6 months) | Antibiotics and related medications (within the first 6 months) | Diseases | Staple Food Type | Smoking | Alcohol intake | Province | Urban /Rural | Area |
| YS1 | YG | 60 | Female | 22.27 | Never | Never | None | Rice | Never | Never | GuangXi | Rural | Areawith high longevity in Guangxi (LA) |
| YS10 | YG | 57 | Male | 25.10 | Never | Never | None | Rice | Never | Ever | GuangXi | Rural | |
| YS11 | YG | 56 | Male | 23.38 | Never | Never | None | Rice | Never | Never | GuangXi | Rural | |
| YS12 | YG | 56 | Male | 20.43 | Never | Never | None | Rice | Never | Never | GuangXi | Rural | |
| YS13 | YG | 55 | Male | 21.50 | Never | Never | None | Rice | Never | Never | GuangXi | Rural | |
| YS14 | YG | 56 | Male | 23.06 | Never | Never | None | Rice | Never | Never | GuangXi | Rural | |
| YS15 | YG | 55 | Male | 21.94 | Never | Never | None | Rice | Never | Never | GuangXi | Rural | |
| YS16 | YG | 55 | Female | 22.63 | Never | Never | None | Rice | Never | Never | GuangXi | Rural | |
| YS17 | YG | 55 | Female | 24.48 | Never | Never | None | Rice | Never | Never | GuangXi | Rural | |
| YS18 | YG | 55 | Male | 21.81 | Never | Never | None | Rice | Never | Never | GuangXi | Rural | |
| YS19 | YG | 62 | Male | 22.06 | Never | Never | None | Rice | Never | Never | GuangXi | Rural | |
| YS2 | YG | 60 | Male | 21.12 | Never | Never | None | Rice | Never | Never | GuangXi | Rural | |
| YS20 | YG | 69 | Male | 23.17 | Never | Never | None | Rice | Never | Never | GuangXi | Rural | |
| YS21 | YG | 66 | Male | 21.03 | Never | Never | None | Rice | Never | Never | GuangXi | Rural | |
| YS22 | YG | 66 | Male | 20.28 | Never | Never | None | Rice | Never | Never | GuangXi | Rural | |
| YS23 | YG | 65 | Male | 24.47 | Never | Never | None | Rice | Never | Never | GuangXi | Rural | |
| YS24 | YG | 64 | Female | 23.50 | Never | Never | None | Rice | Never | Never | GuangXi | Rural | |
| YS25 | YG | 63 | Male | 20.88 | Never | Never | None | Rice& Wheat | Never | Never | GuangXi | Rural | |
| YS26 | YG | 62 | Female | 21.71 | Never | Never | None | Rice | Never | Never | GuangXi | Rural | |
| YS27 | YG | 61 | Female | 23.74 | Never | Never | Hypertersion | Rice | Never | Never | GuangXi | Rural | |
| YS28 | YG | 61 | Male | 20.97 | Never | Never | None | Rice | Never | Eever | GuangXi | Rural | |

| | | | | | | | | | | | | | |
|---|---|---|---|---|---|---|---|---|---|---|---|---|---|
| YS29 | YG | 60 | Male | 23.83 | Never | Never | None | Rice | Never | Eever | GuangXi | Rural | |
| YS3 | YG | 60 | Male | 24.83 | Never | Never | None | Rice | Never | Eever | GuangXi | Rural | |
| YS30 | YG | 60 | Male | 23.84 | Never | Never | None | Rice | Never | Eever | GuangXi | Rural | |
| YS31 | YG | 60 | Male | 24.47 | Never | Never | None | Rice | Never | Never | GuangXi | Rural | |
| YS32 | YG | 58 | Female | 22.48 | Never | Never | None | Rice | Never | Never | GuangXi | Rural | |
| YS33 | YG | 56 | Female | 22.24 | Never | Never | None | Rice | Never | Never | GuangXi | Rural | |
| YS34 | YG | 56 | Female | 25.38 | Never | Never | None | Rice | Never | Never | GuangXi | Rural | |
| YS35 | YG | 56 | Female | 22.30 | Never | Never | None | Rice | Never | Never | GuangXi | Rural | |
| YS36 | YG | 55 | Male | 25.17 | Never | Never | None | Rice | Never | Never | GuangXi | Rural | |
| YS4 | YG | 59 | Male | 24.45 | Never | Never | None | Rice | Never | Never | GuangXi | Rural | |
| YS5 | YG | 58 | Female | 23.71 | Never | Never | None | Rice | Never | Never | GuangXi | Rural | |
| YS6 | YG | 57 | Male | 24.79 | Never | Never | Hypertersion | Rice | Never | Never | GuangXi | Rural | |
| YS7 | YG | 55 | Male | 20.51 | Never | Never | None | Rice | Never | Never | GuangXi | Rural | Areawith high longevity in Guangxi (LA) |
| YS8 | YG | 57 | Female | 23.91 | Never | Never | None | Rice | Never | Never | GuangXi | Rural | |
| YS9 | YG | 57 | Female | 22.82 | Never | Never | None | Rice | Never | Never | GuangXi | Rural | |
| CE1 | LG | 116 | Female | 17.03 | Never | Never | None | Rice& Wheat | Never | Never | GuangXi | Rural | |
| CE10 | LG | 102 | Female | 20.35 | Never | Never | None | Rice | Never | Never | GuangXi | Rural | |
| CE11 | LG | 102 | Female | 19.61 | Never | Never | None | Rice | Never | Never | GuangXi | Rural | |
| CE12 | LG | 101 | Female | 20.62 | Never | Never | None | Rice | Never | Never | GuangXi | Rural | |
| CE13 | LG | 101 | Female | 17.03 | Never | Never | None | Rice | Never | Never | GuangXi | Rural | |
| CE14 | LG | 101 | Female | 18.32 | Never | Never | None | Rice | Never | Never | GuangXi | Rural | |
| CE15 | LG | 101 | Female | 19.32 | Never | Never | None | Rice | Never | Never | GuangXi | Rural | |
| CE16 | LG | 118 | Female | 20.03 | Never | Never | None | Rice | Never | Never | GuangXi | Rural | |
| CE17 | LG | 111 | Female | 19.69 | Never | Never | None | Rice | Never | Never | GuangXi | Rural | |
| CE18 | LG | 108 | Female | 20.47 | Never | Never | None | Rice | Never | Never | GuangXi | Rural | |
| CE37 | LG | 103 | Male | 17.57 | Never | Never | None | Rice | Never | Ever | GuangXi | Rural | |
| CE19 | LG | 108 | Female | 19.51 | Never | Never | None | Rice | Never | Never | GuangXi | Rural | |

| | | | | | | | | | | | | | |
|---|---|---|---|---|---|---|---|---|---|---|---|---|---|
| CE2 | LG | 109 | Female | 19.58 | Never | Never | None | Rice&<br>Wheat | Never | Never | GuangXi | Rural | Areawith high longevity in Guangxi (LA) |
| CE20 | LG | 107 | Female | 18.14 | Never | Never | None | Rice&<br>Wheat | Never | Never | GuangXi | Rural | |
| CE21 | LG | 107 | Female | 17.17 | Never | Never | None | Rice&<br>Wheat | Never | Never | GuangXi | Rural | |
| CE22 | LG | 106 | Female | 17.87 | Never | Never | None | Rice | Never | Never | GuangXi | Rural | |
| CE23 | LG | 104 | Female | 21.20 | Never | Never | Hypertersion | Rice | Never | Never | GuangXi | Rural | |
| CE24 | LG | 104 | Female | 20.41 | Never | Never | None | Rice | Never | Never | GuangXi | Rural | |
| CE25 | LG | 103 | Female | 20.31 | Never | Never | None | Rice | Never | Never | GuangXi | Rural | |
| CE26 | LG | 102 | Female | 18.80 | Never | Never | None | Rice | Never | Never | GuangXi | Rural | |
| CE27 | LG | 102 | Female | 17.20 | Never | Never | None | Rice | Never | Never | GuangXi | Rural | |
| CE28 | LG | 102 | Female | 19.57 | Never | Never | None | Rice | Never | Never | GuangXi | Rural | |
| CE29 | LG | 102 | Female | 20.96 | Never | Never | None | Rice | Never | Never | GuangXi | Rural | |
| CE3 | LG | 108 | Female | 18.69 | Never | Never | Hypertersion | Rice | Never | Never | GuangXi | Rural | |
| CE30 | LG | 102 | Female | 20.75 | Never | Never | None | Rice | Never | Never | GuangXi | Rural | |
| CE31 | LG | 101 | Female | 18.06 | Never | Never | None | Rice | Never | Never | GuangXi | Rural | |
| CE32 | LG | 101 | Female | 17.60 | Never | Never | None | Rice | Never | Never | GuangXi | Rural | |
| CE33 | LG | 108 | Female | 19.42 | Never | Never | None | Rice&<br>Wheat | Never | Never | GuangXi | Rural | |
| CE34 | LG | 108 | Female | 17.87 | Never | Never | None | Rice | Never | Never | GuangXi | Rural | |
| CE35 | LG | 105 | Female | 19.47 | Never | Never | None | Rice | Never | Never | GuangXi | Rural | |
| CE36 | LG | 104 | Male | 19.95 | Never | Never | None | Rice | Never | Ever | GuangXi | Rural | |
| CE38 | LG | 103 | Female | 18.74 | Never | Never | None | Rice | Never | Never | GuangXi | Rural | |
| CE39 | LG | 101 | Female | 17.60 | Never | Never | None | Rice | Never | Never | GuangXi | Rural | |
| CE4 | LG | 105 | Female | 19.56 | Never | Never | None | Rice | Never | Never | GuangXi | Rural | |
| CE5 | LG | 105 | Female | 21.03 | Never | Never | Hypertersion | Rice | Never | Never | GuangXi | Rural | |
| CE6 | LG | 105 | Female | 21.05 | Never | Never | None | Rice | Never | Never | GuangXi | Rural | |

| | | | | | | | | | | | | | |
|---|---|---|---|---|---|---|---|---|---|---|---|---|---|
| CE7 | LG | 104 | Female | 19.30 | Never | Never | None | Rice | Never | Never | GuangXi | Rural | |
| CE8 | LG | 102 | Female | 21.02 | Never | Never | None | Rice | Never | Never | GuangXi | Rural | |
| CE9 | LG | 102 | Female | 20.78 | Never | Never | None | Rice | Never | Never | GuangXi | Rural | |
| GE1 | YG | 86 | Male | 23.41 | Never | Never | None | Rice | Never | Never | GuangXi | Rural | |
| GE10 | YG | 73 | Male | 23.84 | Never | Never | None | Rice | Never | Never | GuangXi | Rural | |
| GE11 | YG | 73 | Female | 24.69 | Never | Never | None | Rice | Never | Never | GuangXi | Rural | |
| GE12 | YG | 73 | Male | 21.59 | Never | Never | None | Rice& Wheat | Never | Never | GuangXi | Rural | |
| GE13 | YG | 72 | Male | 23.03 | Never | Never | None | Rice | Ever | Never | GuangXi | Rural | |
| GE14 | YG | 71 | Female | 21.65 | Never | Never | None | Rice | Never | Ever | GuangXi | Rural | |
| GE15 | YG | 71 | Male | 24.50 | Never | Never | None | Rice | Never | Ever | GuangXi | Rural | |
| GE16 | YG | 71 | Male | 24.45 | Never | Never | None | Rice& Wheat | Never | Never | GuangXi | Rural | Areawith high longevity in Guangxi (LA) |
| GE17 | YG | 80 | Female | 24.81 | Never | Never | None | Rice | Never | Never | GuangXi | Rural | |
| GE18 | YG | 76 | Female | 23.92 | Never | Never | None | Rice | Never | Never | GuangXi | Rural | |
| GE19 | YG | 72 | Female | 24.87 | Never | Never | None | Rice | Never | Never | GuangXi | Rural | |
| GE2 | YG | 86 | Male | 23.60 | Never | Never | None | Rice | Never | Never | GuangXi | Rural | |
| GE20 | YG | 86 | Female | 22.57 | Never | Never | None | Rice | Never | Never | GuangXi | Rural | |
| GE21 | YG | 85 | Female | 20.95 | Never | Never | Hypertersion | Rice | Never | Never | GuangXi | Rural | |
| GE3 | YG | 85 | Female | 20.73 | Never | Never | Hypertersion | Rice | Never | Never | GuangXi | Rural | |
| GE4 | YG | 84 | Male | 21.02 | Never | Never | None | Rice | Never | Never | GuangXi | Rural | |
| GE5 | YG | 80 | Male | 22.99 | Never | Never | None | Rice | Never | Never | GuangXi | Rural | |
| GE6 | YG | 79 | Female | 20.01 | Never | Never | None | Rice | Never | Never | GuangXi | Rural | |
| GE7 | YG | 76 | Female | 18.78 | Never | Never | None | Rice | Never | Never | GuangXi | Rural | |
| GE8 | YG | 75 | Female | 22.03 | Never | Never | None | Rice | Never | Never | GuangXi | Rural | |
| GE9 | YG | 73 | Male | 19.61 | Never | Never | None | Rice | Never | Never | GuangXi | Rural | |
| OS1 | LG | 98 | Female | 21.38 | Never | Never | None | Rice | Never | Never | GuangXi | Rural | |
| OS10 | LG | 92 | Female | 17.23 | Never | Never | None | Rice | Never | Never | GuangXi | Rural | |

| | | | | | | | | | | | | | |
|---|---|---|---|---|---|---|---|---|---|---|---|---|---|
| OS11 | LG | 91 | Male | 19.14 | Never | Never | None | Rice | Never | Never | GuangXi | Rural | |
| OS12 | LG | 91 | Male | 16.24 | Never | Never | None | Rice& Wheat | Never | Never | GuangXi | Rural | |
| OS13 | LG | 100 | Female | 18.33 | Never | Never | Hypertersion | Rice | Never | Never | GuangXi | Rural | |
| OS14 | LG | 100 | Male | 20.60 | Never | Never | None | Rice& Wheat | Never | Never | GuangXi | Rural | |
| OS15 | LG | 100 | Male | 20.69 | Never | Never | None | Rice | Never | Never | GuangXi | Rural | |
| OS16 | LG | 98 | Female | 16.33 | Never | Never | None | Rice | Never | Never | GuangXi | Rural | |
| OS17 | LG | 96 | Female | 18.09 | Never | Never | None | Rice | Never | Never | GuangXi | Rural | |
| OS18 | LG | 95 | Female | 17.43 | Never | Never | None | Rice | Never | Never | GuangXi | Rural | |
| OS19 | LG | 94 | Male | 20.07 | Never | Never | None | Rice | Never | Never | GuangXi | Rural | |
| OS2 | LG | 98 | Female | 21.44 | Never | Never | None | Rice | Never | Never | GuangXi | Rural | Areawith high longevity in Guangxi (LA) |
| OS20 | LG | 93 | Male | 18.29 | Never | Never | None | Rice | Never | Never | GuangXi | Rural | |
| OS21 | LG | 93 | Female | 21.08 | Never | Never | None | Rice | Never | Never | GuangXi | Rural | |
| OS22 | LG | 93 | Female | 19.02 | Never | Never | None | Rice | Never | Never | GuangXi | Rural | |
| OS23 | LG | 93 | Female | 17.40 | Never | Never | None | Rice | Never | Never | GuangXi | Rural | |
| OS24 | LG | 92 | Female | 19.62 | Never | Never | None | Rice | Never | Never | GuangXi | Rural | |
| OS25 | LG | 92 | Female | 21.02 | Never | Never | None | Rice | Never | Never | GuangXi | Rural | |
| OS26 | LG | 92 | Female | 19.16 | Never | Never | None | Rice | Never | Never | GuangXi | Rural | |
| OS27 | LG | 92 | Female | 15.91 | Never | Never | None | Rice | Never | Never | GuangXi | Rural | |
| OS28 | LG | 91 | Male | 17.80 | Never | Never | None | Rice | Never | Never | GuangXi | Rural | |
| OS29 | LG | 91 | Female | 18.33 | Never | Never | None | Rice | Never | Never | GuangXi | Rural | |
| OS3 | LG | 96 | Female | 17.80 | Never | Never | None | Rice& Wheat | Never | Never | GuangXi | Rural | |
| OS30 | LG | 91 | Female | 16.90 | Never | Never | None | Rice& Wheat | Never | Never | GuangXi | Rural | |
| OS31 | LG | 91 | Female | 17.03 | Never | Never | None | Rice& Wheat | Never | Never | GuangXi | Rural | |

| | | | | | | | | | | | | | |
|---|---|---|---|---|---|---|---|---|---|---|---|---|---|
| OS32 | LG | 91 | Female | 20.13 | Never | Never | None | Rice | Never | Never | GuangXi | Rural | Areawith high longevity in Guangxi (LA) |
| OS33 | LG | 90 | Female | 21.37 | Never | Never | None | Rice | Never | Never | GuangXi | Rural | |
| OS34 | LG | 100 | Male | 18.76 | Never | Never | None | Rice | Never | Never | GuangXi | Rural | |
| OS35 | LG | 99 | Male | 17.16 | Never | Never | None | Rice | Never | Never | GuangXi | Rural | |
| OS36 | LG | 98 | Female | 16.67 | Never | Never | None | Rice | Never | Never | GuangXi | Rural | |
| OS37 | LG | 95 | Female | 18.87 | Never | Never | None | Rice | Never | Never | GuangXi | Rural | |
| OS38 | LG | 95 | Male | 18.91 | Never | Never | None | Rice | Never | Never | GuangXi | Rural | |
| OS39 | LG | 90 | Female | 21.08 | Never | Never | None | Rice | Never | Never | GuangXi | Rural | |
| OS4 | LG | 96 | Female | 16.41 | Never | Never | None | Rice | Never | Never | GuangXi | Rural | |
| OS5 | LG | 94 | Female | 21.36 | Never | Never | None | Rice | Never | Never | GuangXi | Rural | |
| OS6 | LG | 93 | Female | 17.22 | Never | Never | None | Rice | Never | Never | GuangXi | Rural | |
| OS7 | LG | 92 | Female | 16.95 | Never | Never | None | Rice | Never | Never | GuangXi | Rural | |
| OS8 | LG | 92 | Female | 20.77 | Never | Never | None | Rice | Never | Never | GuangXi | Rural | |
| OS9 | LG | 92 | Female | 17.74 | Never | Never | None | Rice | Never | Never | GuangXi | Rural | |
| NLA1 | NLA | 61 | Female | 22.11 | Never | Never | None | Rice | Never | Never | GuangXi | Urban | Area with low longevity in Guangxi (NLA) |
| NLA2 | NLA | 61 | Male | 24.36 | Never | Never | None | Rice | Never | Never | GuangXi | Urban | |
| NLA3 | NLA | 63 | Male | 20.94 | Never | Never | None | Rice | Ever | Never | GuangXi | Urban | |
| NLA4 | NLA | 64 | Male | 20.67 | Never | Never | None | Rice | Never | Never | GuangXi | Urban | |
| NLA5 | NLA | 65 | Male | 23.40 | Never | Never | None | Rice | Never | Never | GuangXi | Urban | |
| NLA6 | NLA | 66 | Female | 20.66 | Never | Never | None | Rice | Never | Never | GuangXi | Urban | |
| NLA7 | NLA | 70 | Male | 23.15 | Never | Never | None | Rice | Never | Never | GuangXi | Urban | |
| NLA8 | NLA | 70 | Female | 21.47 | Never | Never | None | Rice | Never | Never | GuangXi | Urban | |
| NLA9 | NLA | 71 | Male | 20.51 | Never | Never | None | Rice | Ever | Never | GuangXi | Urban | |
| NLA10 | NLA | 72 | Female | 23.88 | Never | Never | None | Rice | Never | Ever | GuangXi | Urban | |
| NLA11 | NLA | 80 | Male | 24.06 | Never | Never | Hypertersion | Rice | Never | Never | GuangXi | Urban | |
| NLA12 | NLA | 82 | Female | 19.86 | Never | Never | Hypertersion | Rice | Never | Never | GuangXi | Urban | |
| NLA13 | NLA | 53 | Male | 24.61 | Never | Never | None | Rice | Never | Never | GuangXi | Urban | |
| NLA14 | NLA | 54 | Female | 20.00 | Never | Never | None | Rice | Never | Never | GuangXi | Urban | |

| | | | | | | | | | | | | | |
|---|---|---|---|---|---|---|---|---|---|---|---|---|---|
| NLA15 | NLA | 54 | Female | 22.06 | Never | Never | None | Rice | Never | Never | GuangXi | Urban | |
| NLA16 | NLA | 55 | Male | 25.22 | Never | Never | None | Rice | Never | Ever | GuangXi | Urban | |
| NLA17 | NLA | 56 | Male | 24.42 | Never | Never | None | Rice | Never | Never | GuangXi | Urban | |
| NLA18 | NLA | 56 | Female | 22.34 | Never | Never | None | Rice | Ever | Never | GuangXi | Urban | NLA |
| NLA19 | NLA | 56 | Male | 19.90 | Never | Never | None | Rice | Never | Never | GuangXi | Urban | |
| NLA20 | NLA | 56 | Male | 25.20 | Never | Never | None | Rice | Never | Ever | GuangXi | Urban | |
| NLA21 | NLA | 59 | Female | 22.11 | Never | Never | None | Rice | Never | Never | GuangXi | Urban | |
| NLA22 | NLA | 59 | Female | 20.42 | Never | Never | None | Rice | Never | Never | GuangXi | Urban | |

**Table S2.** Primers and conditions.

| ID | Primer Sequence (5′-3′) | Annealing Temperature (℃) |
|---|---|---|
| Total Bacteria | F: ACTCCTACGGCAGGCAGCAGT<br>R: ATTACCGCGGCTGCTGGC | 65 |
| *Alistipes* | F: CTATGCCGTGCCGTTCGATACC<br>R: AGTAATCCTTGTCGTCCTCCTCCTG | 60 |
| *Bacteroides* | F: GTCACACTCAGTCACCGACAACAC<br>R: GAGCACGGATACTCTGAACAGCATC | 60 |
| *Blautia* | F: GTCACACTCAGTCACCGACAACAC<br>R: GAGCACGGATACTCTGAACAGCATC | 60 |
| *Lachnospiraceae bacterium NK4A136* | F: AGATCAGAATGCTGCGGTGAATACG<br>R: CCCAGTTATCAATCCTGCCTTCGG | 62 |
| *Lactobacillus* | F: AGATCAGAATGCTGCGGTGAATACG<br>R: CCCAGTTATCAATCCTGCCTTCGG | 62 |
| 27F | AGAGTTTGATCCTGGCCTCA | 55 |
| 1492R | GGTTACCTTGTTTGTTACGACTT | 55 |

**Table S3.** PCR reaction system for strain identification.

| Reaction System | Volume ( μ L) |
|---|---|
| 2 × Master Mix | 12.50 |
| Primer 1 | 0.75 |
| Primer 2 | 0.75 |
| DNA template | 1.00 |
| $ddH_2O$ | 10.00 |

**Table S4.** PCR conditions system for strain identification.

| Temperature (℃) | Reaction | Reaction time | Number of cycles |
|---|---|---|---|
| 94 | pre-denaturation | 3 min | 1 |
| 94 | denaturation | 30 s | 30 |
| 55 | Annealing | 30 s | 30 |
| 72 | Extension | 1 min | 30 |
| 72 | Re-extension | 5 min | 1 |

**Table S5.** Size of each module.Top 10 in the mediumpurple3 network ranked by MNC method.

| Rank | OTU_ID | Taxonomy |
|---|---|---|
| 1 | OTU_46016 | *Bacteria; Firmicutes; Clostridia; Clostridiales; Lachnospiraceae; Blautia; uncultured bacterium* |
| 2 | OTU_45904 | *Bacteria; Firmicutes; Bacilli; Lactobacillales; Lactobacillaceae; Lactobacillus; uncultured bacterium* |
| 2 | OTU_46034 | *Bacteria; Firmicutes; Clostridia; Clostridiales; Ruminococcaceae; [Eubacterium] coprostanoligenes group; uncultured bacterium* |
| 4 | OTU_46057 | *Bacteria; Firmicutes; Clostridia; Clostridiales; Christensenellaceae; Christensenellaceae R-7 group; uncultured bacterium* |
| 4 | OTU_46004 | *Bacteria; Bacteroidetes; Bacteroidia; Bacteroidales; Bacteroidaceae; Bacteroides; uncultured bacterium* |
| 4 | OTU_45797 | *Bacteria; Bacteroidetes; Bacteroidia; Bacteroidales; Rikenellaceae; Alistipes; Alistipes finegoldii DSM 17242* |
| 4 | OTU_45857 | *Bacteria; Actinobacteria; Actinobacteria; Bifidobacteriales; Bifidobacteriaceae; Bifidobacterium; Bifidobacterium longum NCC2705* |
| 8 | OTU_45834 | *Bacteria; Firmicutes; Clostridia; Clostridiales; Ruminococcaceae; Ruminiclostridium 5; uncultured bacterium* |
| 8 | OTU_46033 | *Bacteria; Firmicutes; Clostridia; Clostridiales; Lachnospiraceae; Lachnospiraceae NK4A136 group; uncultured bacterium* |
| 10 | OTU_45907 | *Bacteria; Firmicutes; Clostridia; Clostridiales; Ruminococcaceae; Ruminococcaceae UCG-004; uncultured bacterium* |

**Table S6.** Basic characteristics of the strains.

| | **Survival rate after simulated gastrointestinal fluid transfer(%)** | **Self-agglutination rate (5h, %)** | **Percentage of adhesion (%)** |
|---|---|---|---|
| *Lactobacillus fermentum* LTP1806 | 67.65 ± 0.06 | 76.89 ± 2.52 | 76.13 ± 1.10 |
| *Lactobacillus fermentum* LTP1805 | 6.53 ± 0.02 | 64.68 ± 0.72 | 59.54 ± 1.43 |
| *Lactobacillus fermentum* LTP1804 | 9.09 ± 0.00 | 57.96 ± 9.50 | 15.82 ± 0.37 |
| *Lactobacillus fermentum* LTP1334 | 88.92 ± 1.26 | 81.34 ± 0.13 | 76.68 ± 0.02 |
| *Lactobacillus fermentum* LTP1332 | 90.23 ± 2.86 | 78.88 ± 0.01 | 81.77 ± 0.02 |
| *Lactobacillus fermentum* LTP1333 | 91.29 ± 0.85 | 71.50 ± 0.72 | 67.06 ± 0.80 |
| *Lactobacillus fermentum* LTP1848 | 30.51 ± 0.45 | 63.04 ± 6.43 | 55.43 ± 1.14 |
| *Lactobacillus fermentum* LTP1832 | 72.58 ± 0.15 | 72.63 ± 0.32 | 42.60 ± 6.55 |
| *Bacteroides fragilis* LTBF34 | 33.75 ± 1.04 | 35.04 ± 0.07 | 22.29 ± 0.11 |
| *Bacteroides fragilis* LTBF32 | 35.62 ± 4.58 | 36.71 ± 0.48 | 23.18 ± 0.53 |
| *Bacteroides fragilis* LTBF31 | 55.88 ± 1.27 | 43.98 ± 0.92 | 45.75 ± 0.03 |
| *Bacteroides fragilis* LTBF13 | 64.46 ± 0.55 | 58.67 ± 0.21 | 56.08 ± 0.58 |
| *Bacteroides fragilis* LTBF12 | 79.07 ± 2.67 | 51.60 ± 0.52 | 64.97 ± 0.30 |
| *Bacteroides fragilis* LTBF11 | 68.00 ± 3.46 | 56.51 ± 2.08 | 44.07 ± 0.26 |

All values are presented as mean (SD).

## Figures

**Figure S1.** Diagram showing experimental design and animal groupings for mice.

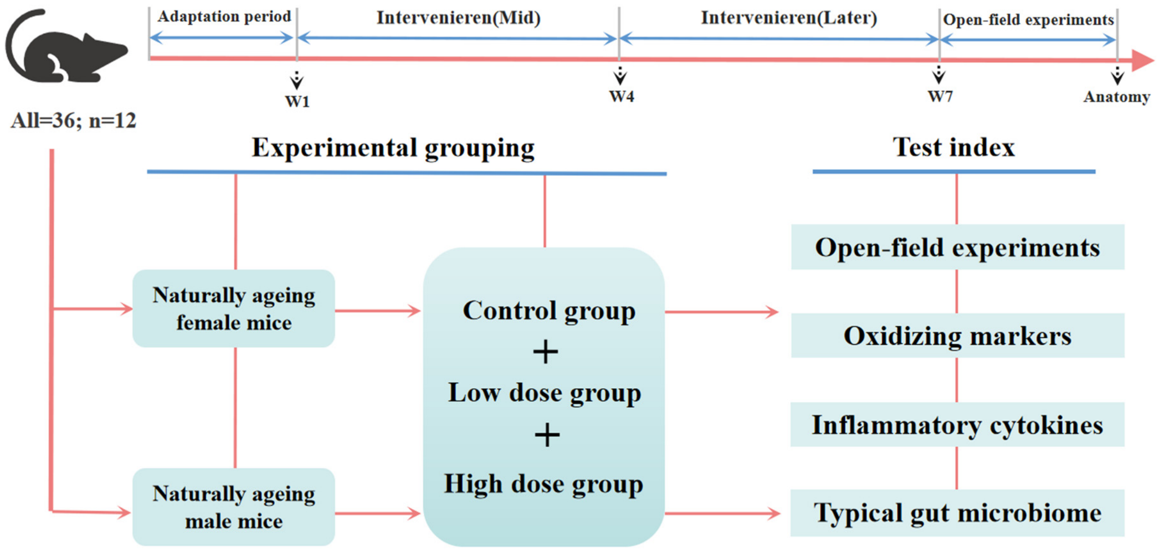


**Figure S1.** After one week of acclimatization feeding, mice were randomly divided into three groups: control (C) mice were gavaged with sterile saline (same volume as used in the probiotic combination) daily; low-dose (L) group was gavaged with 1 × $10^7$ CFU/mL *Lactobacillus fermentum* LTP1332 + 1 × $10^7$ CFU/mL *Bacteroides fragilis* LTBF12 daily; high-dose (H) group was gavaged with 1 × $10^9$ CFU/mL *Lactobacillus fermentum* LTP1332 + 1 × $10^9$ CFU/mL *Bacteroides fragilis* LTBF12 daily, 12 males and females in each group for 6 weeks. The overall intervention process is shown in the diagram below.

**Figure S2.** Structural characteristics of the microbiota.

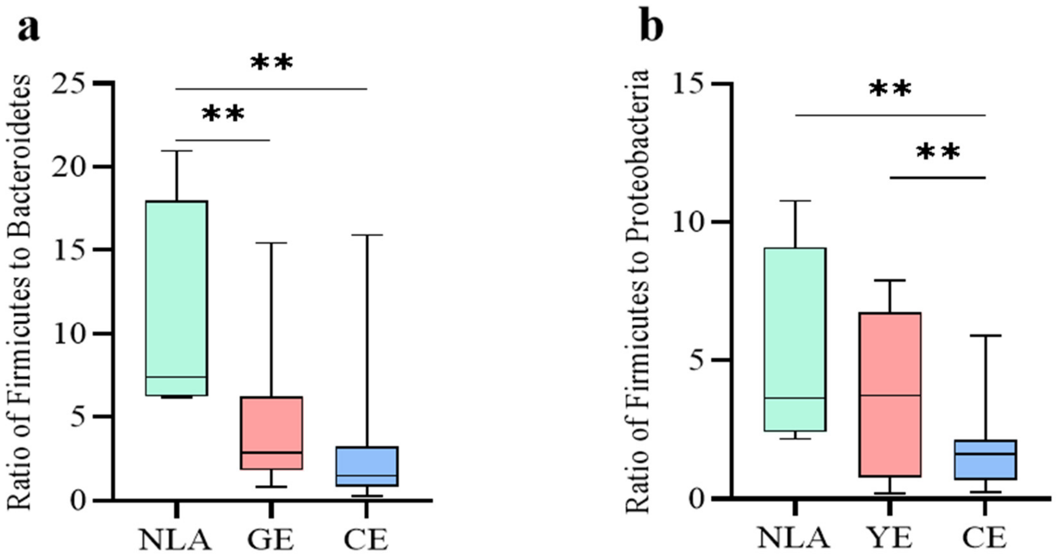


**Figure S2.** Ratio of typical bacterial phylum in the centenarian (CE) to the general elderly (GE) in the longevity area and the 70-86 year old group in the non-longevity area (NLA). (**a**) Indicates the ratio of *Firmicutes* to *Bacteroides* (F/B); (**b**) Indicates the ratio of *Firmicutes* to *Proteobacteria* (F/P). ** indicates $p < 0.01$ (Wilcoxon rank sum test).

**Figure S3.** The selection process of a soft threshold.

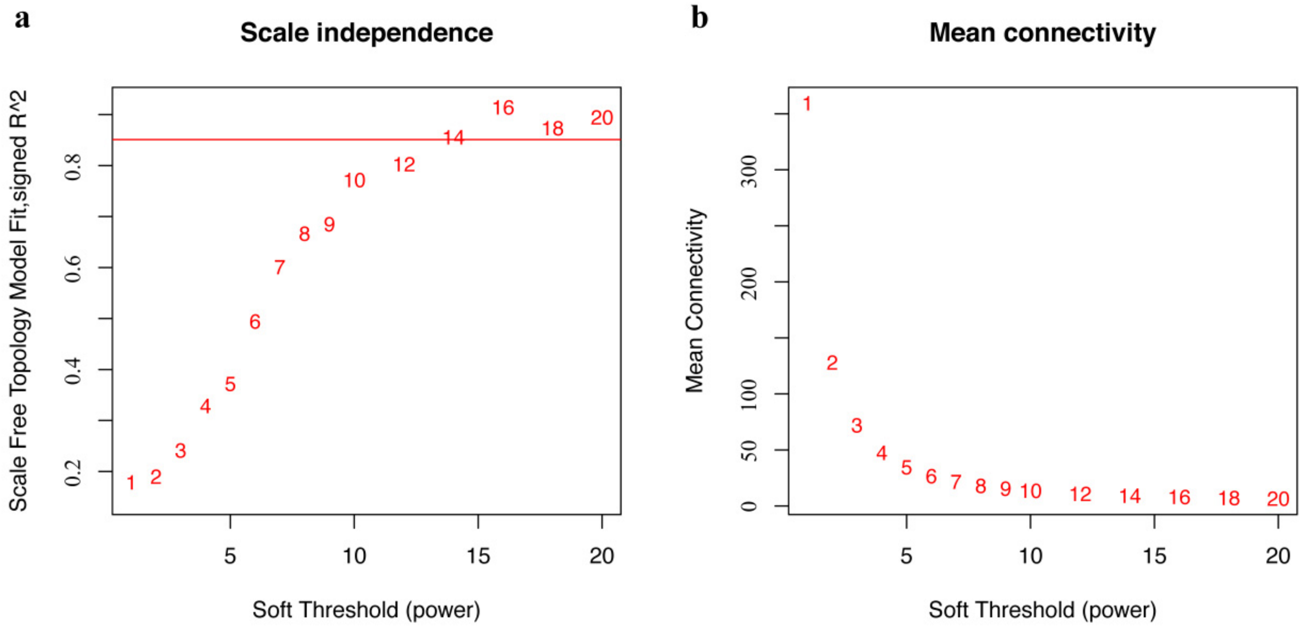


**Figure S3.** (**a**) The abscissa represents the value of the power index, and the ordinate represents the scale-free topological fitting index $R^2$. When $R^2$ is close to 0.85, the established network is closer to the scale-free network. (b) The abscissa represents the value of the power index, and the ordinate represents the corresponding average number of left and right nodes. The average connectivity gradually approaches zero, indicating that the average connectivity of the network is good.

**Figure S4.** Size of each module.

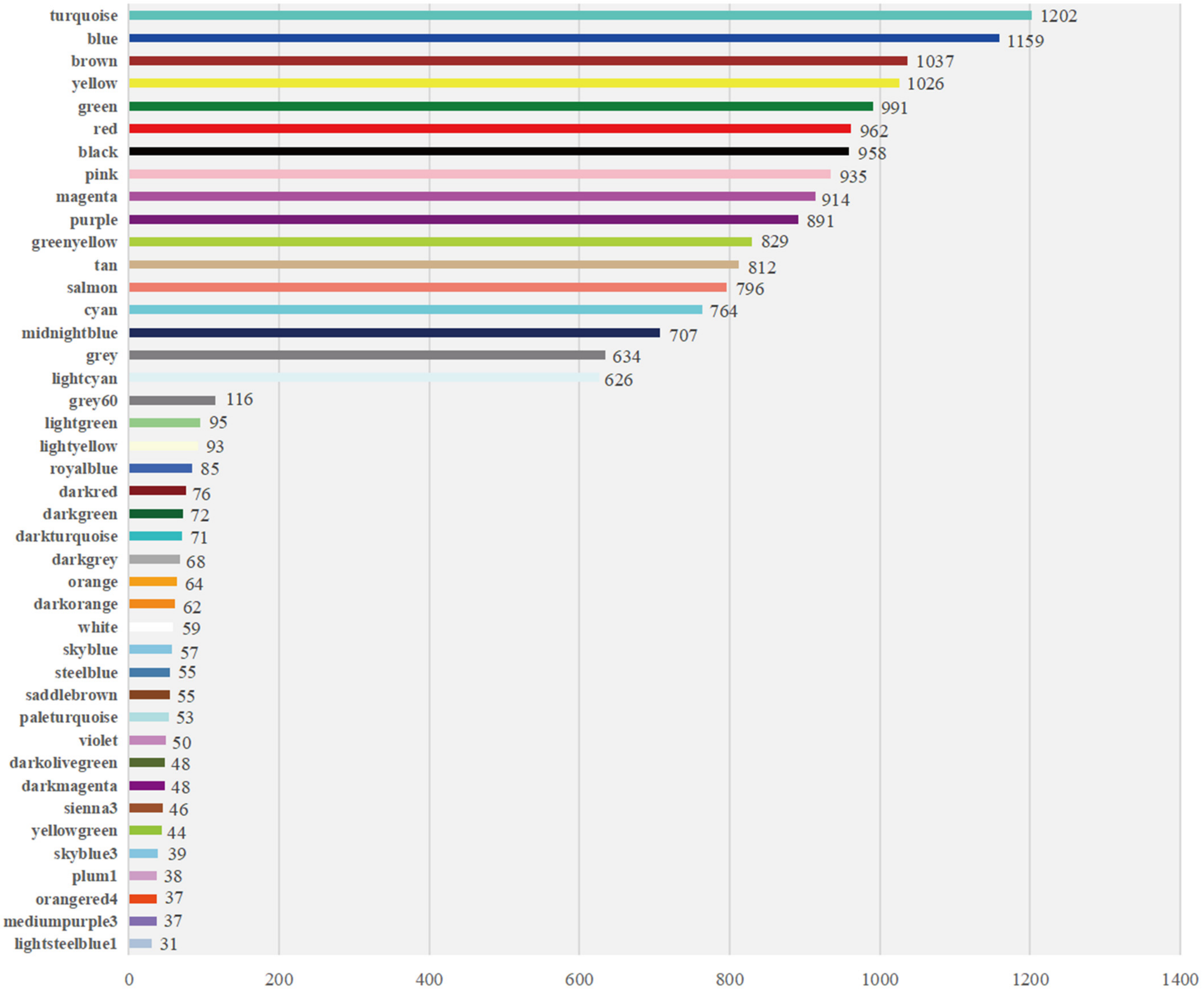


**Figure S4.** The bar graph with different colours reflects the number of OTU clusters in different modules. The number of clusters in the module varies from 31 to 1202. Of all the taxons, only 634 taxons (3.79%) are not included in any colour module. These taxons are classified into the grey module by default.

**Figure S5.** Functional prediction results.

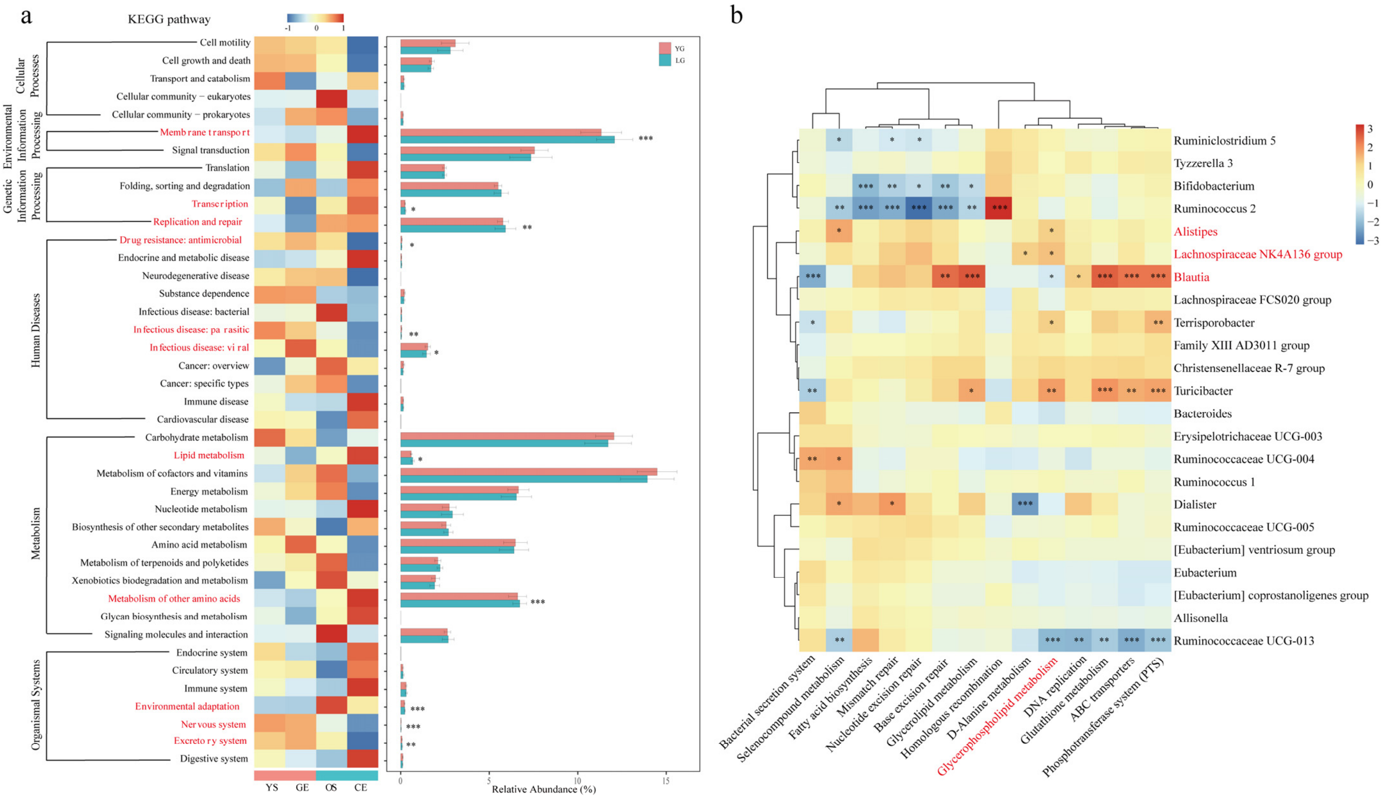


**Figure S5.** Functional predictions within key modules. (**a**) Functional predictions of the first versus second cascade and results of inter-group variance analysis. The left side of the image depicts the biological function prediction of the intestinal flora in the Guangxi longevity zone based on the KEGG database, where the first-tier metabolic pathway and the second-tier metabolic pathway of KEGG are included. The right side of the image depicts the relative abundance and differences in KEGG second-level metabolic pathways between the LG and YG groups. A heat map showing the correlation between the relative abundance of KEGG second-level metabolic pathways and each age grouping is shown in the middle of the image. (**b**) Heat map of the correlation between the abundance of the tertiary metabolic pathways with significant differences of >1% and the abundance of the genera within the module, calculated using Pearson. * indicates $p < 0.05$, ** indicates $p < 0.01$ and *** indicates $p < 0.001$ (Wilcoxon rank sum test).
